# Feedback between mechanosensitive signaling and active forces governs endothelial junction integrity

**DOI:** 10.1038/s41467-022-34701-y

**Published:** 2022-11-19

**Authors:** Eoin McEvoy, Tal Sneh, Emad Moeendarbary, Yousef Javanmardi, Nadia Efimova, Changsong Yang, Gloria E. Marino-Bravante, Xingyu Chen, Jorge Escribano, Fabian Spill, José Manuel Garcia-Aznar, Ashani T. Weeraratna, Tatyana M. Svitkina, Roger D. Kamm, Vivek B. Shenoy

**Affiliations:** 1 Department of Materials Science and Engineering, University of Pennsylvania, Philadelphia, PA 19104 USA; 2 Center for Engineering Mechanobiology, University of Pennsylvania, Philadelphia, PA 19104 USA; 3 Biomedical Engineering, University of Galway, Galway, H91 HX31 Ireland; 4 Center for Molecular Design and Biomimetics, The Biodesign Institute, Arizona State University, Tempe, AZ 85287 USA; 5 Department of Mechanical Engineering, University College London, London, WC1E 7JE UK; 6 Department of Biological Engineering, Massachusetts Institute of Technology, Cambridge, MA 02139 USA; 7 Department of Biology, University of Pennsylvania, Philadelphia, PA 19104 USA; 8 Department of Biochemistry and Molecular Biology, Johns Hopkins Bloomberg School of Public Health, Baltimore, MD 21205 USA; 9 Department of Oncology, Sidney Kimmel Cancer Center, Johns Hopkins School of Medicine, Baltimore, MD 21205 USA; 10 Department of Mechanical Engineering, University of Zaragoza, Zaragoza, Spain; 11 School of Mathematics, University of Birmingham, Birmingham, B15 2TT United Kingdom; 12 Department of Mechanical Engineering, Massachusetts Institute of Technology, Cambridge, MA 02139 USA

**Keywords:** Computational models, Biophysics, Computational biophysics, Cell adhesion, Cytoskeleton

## Abstract

The formation and recovery of gaps in the vascular endothelium governs a wide range of physiological and pathological phenomena, from angiogenesis to tumor cell extravasation. However, the interplay between the mechanical and signaling processes that drive dynamic behavior in vascular endothelial cells is not well understood. In this study, we propose a chemo-mechanical model to investigate the regulation of endothelial junctions as dependent on the feedback between actomyosin contractility, VE-cadherin bond turnover, and actin polymerization, which mediate the forces exerted on the cell-cell interface. Simulations reveal that active cell tension can stabilize cadherin bonds, but excessive RhoA signaling can drive bond dissociation and junction failure. While actin polymerization aids gap closure, high levels of Rac1 can induce junction weakening. Combining the modeling framework with experiments, our model predicts the influence of pharmacological treatments on the junction state and identifies that a critical balance between RhoA and Rac1 expression is required to maintain junction stability. Our proposed framework can help guide the development of therapeutics that target the Rho family of GTPases and downstream active mechanical processes.

## Abstract (web summary)

Gap formation in the vasculature underpins immune and tumour cell infiltration. Here the authors propose a chemo-mechanical model to analyse how feedback between mechanosensitive signalling, active cellular forces and adhesion governs the breakdown, recovery, and integrity of endothelial junctions.

## Introduction

The maintenance and turnover of endothelial junctions govern a wide range of vascular activity, from capillary barrier function to vessel branching during angiogenesis^1^. Vascular homeostasis is closely tied to the regulation of this complex system, with junction breakdown mediating leukocyte migration, bruising, and edema^2,3^, in addition to pathological events such as hemorrhagic stroke^4^, onset of atherosclerosis^5^, and tumor cell invasion^6^. As such, endothelial junctions undergo frequent dynamic rearrangements to modify their structure and organization. Cell-cell adhesion is maintained predominantly by adherens junctions (AJs), comprised of cadherin bonds which interface with the actomyosin network and whose stability is tension-regulated^7,8^. Cadherin is therefore essential to normal endothelium activity as, for example, blocking VE-cadherin function using the BV13 antibody completely disassembled blood vessels in mice^9^, and cadherin knockout led to loss of barrier function^10,11^. Further, cadherin endocytosis is a mechanism by which vascular endothelial growth factor (VEGF) can form gaps in existing blood vessels to permit the growth of new capillaries^12^. Unbound cadherin, as a transmembrane protein, is free to diffuse along the membrane, but typically it preferentially localizes at cell-cell contacts^13^. Adhesions at these contacts stabilize and reinforce under tension^14^, establishing a feedback loop with the actomyosin cables. These key cadherin interactions are also calcium dependent and strengthen under the influx of Ca^2+^ from mechanosensitive ion channels^15^.

Forces acting on intercellular junctions trigger a range of biochemical processes that activate the Src family of kinases (SFK), which downstream interact with Rho-GTPases to drive changes in myosin motor activity and cell contractility^16^. Upregulation in RhoA activity can initiate high engagement of myosin with actin filaments to yield dense stress fibers, which can promote clustering of cadherin proteins and transmit high tension to cadherin bonds^17,18^. Mechanical stress regulates cadherin turnover at cell junctions^19^ and directly increases the size of cell-cell adhesions^20,21^. RhoA activation has also been shown to induce junction contraction and strengthening^22,23^. However, RhoA activation via treatment with thrombin has also been reported to cause poor endothelial barrier integrity and junction permeability^24–27^; thus thrombin in relatively high doses is often considered a destabilizing agent^28^. In apparent contradiction, blocking myosin II function via blebbistatin also leads to decreased junctional E-cadherin^29^ and junction breakdown^30^. Interestingly, inhibiting ROCK (kinase activated by RhoA) with Y-27632 is additionally detrimental to junction integrity^30,31^. Another member of the Rho GTPase family, Rac1, is also frequently implicated in the regulation of vascular function and endothelium homeostasis. Rac1 activates the WAVE regulatory complex to drive Arp2/3-associated polymerization of branched actin networks at the cell boundary. Abu Taha and Schnittler (2014) observed that Rac1 induction facilitates the bridging of intercellular gaps and formation of stable junction contacts^1^. Further, junction integrity cannot be maintained following blockage of Arp2/3^30^. By inducing junction tearing, Rajput et al. (2013) demonstrated that with decreasing Rac1 expression, gap closure was increasingly impaired^32^. Conversely, significant upregulation of the Rac1 pathway via platelet-activating factor apparently causes widespread barrier disruption and cadherin endocytosis^11,33^.

The effects of both RhoA and Rac1 signaling are seemingly contradictory across the literature, promoting both junction strengthening and breakdown. Motivated by the gap in knowledge, here we explore the formation, stability, and failure of endothelial contacts and the influence of RhoA and Rac1 signaling pathways on endothelial junction dynamics. We first propose a one-dimensional theoretical model to describe the three-way chemo-mechanical feedback between (i) VE-cadherin bond turnover, (ii) RhoA-mediated actomyosin contractility, and (iii) Rac1-drived actin polymerization, to uncover how endothelial gaps dynamically form and recover. We expand this framework to the analysis of a two-dimensional monolayer, and present insight into the contradictory influence of RhoA and Rac1 expression on junction stability. The model suggests how modulation of signaling and downstream actin polymerization and cell contractility can influence junction stability, which we then compare with the behavior of endothelial cells (HUVECs) under pharmacological treatment with thrombin, Y-27632, PAF, and CK-666. Ultimately, our model indicates that a balance and crosstalk between RhoA and Rac1 signaling could be essential to the regulation of junctional homeostatic function.

## Results

### Endothelial junction integrity is regulated by remodeling of the actin cytoskeleton

**Fig. 1:**
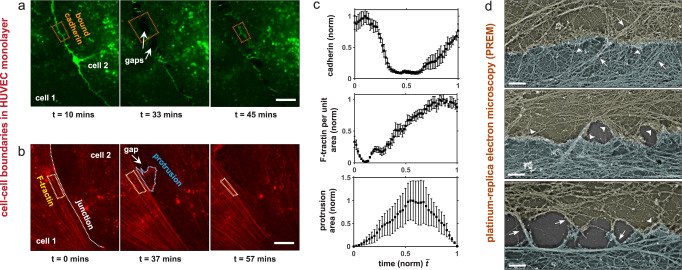
Remodeling of endothelial junctions. **a**, **b** Dynamics of gap formation and recovery in remodeling adherens junctions in HUVECS over time showing (a) GFP-VE-cadherin and (**b**) F-tractin highlighting actin bundles and actin-rich protrusions. Scale bars, 10 $\mu m$; (**c**) Local intensity of cadherin ($n=6$ junctions), F-tractin ($n=4$ junctions), and protrusion area ($n=4$ junctions) along cell-boundaries (mean $\pm$ s.e.m.) in the vicinity of gaps (dashed outlines show examples of areas used for intensity measurements) over time normalized to gap opening (t = 0) and closing (t = 1); **d**) PREM images showing cytoskeleton organization at junctions. Arrows and arrowheads mark stress-fiber-like actin bundles and branch networks, respectively. Scale bars, 1 $\mu m$. Contacting cells are lightly pseudo-colored to highlight cell boundary, which was identified based on immunogold VE-cadherin labeling.

To explore the formation, stability, and disruption of endothelial junctions, we plated human umbilical vascular endothelial cells (HUVECs) on collagen-coated glass-bottom dishes (see Methods). Live-cell imaging of GFP–VE-cadherin and F-tractin (a genetically encoded reporter for F-actin) was performed to track gap formation and recovery at cell-cell interfaces. We observed a high cadherin intensity at the cohesive cell-cell interface, with cadherin concentrations reducing when gaps formed between adhered cells (Fig. 1a–c; Supplementary Movie 1). In regions where junctions broke down and cells retracted, thus forming a gap, actin-rich protrusions subsequently developed locally to restore cell-cell contact (Fig. 1b-c; Supplementary Movies 1-2). Junction recovery corresponded with both an increase in local VE-cadherin at the cell-cell boundary and F-tractin concentrations in bundles located next to the restoring junction (Fig. 1c). Our observed accumulation of bundled actin concurrent with junction restoration indicates an increase in actomyosin contractility. To verify this, staining for myosin (NMII) showed significant co-localization with aligned and contracted actin bundles near adherens junctions (Fig. S5a-c). We next analyzed the structure of the cytoskeleton at cell-cell interfaces by platinum replica electron microscopy (PREM), with cell boundaries identified based on immunogold VE-cadherin labeling. The organization of the cytoskeleton appeared to indicate the junction state at different stages of gap formation and junction recovery (Fig. 1d). Continuous cohesion of two neighboring cells corresponded with the presence of branched actin networks at the cell-cell junction and actin filament bundles (perpendicular, oblique or parallel) in the junction vicinity. Quantification of cell-cell contacts in PREM images (Table S1) revealed that on average 95.24$\pm$12.38% of the length of linear adherens junctions was associated with branched actin networks, whereas branched actin networks were found only occasionally at the cell boundaries facing an intercellular gap (on average, only 18.62 ± 13.26% of gap sides contained any detectable amount of branched actin networks) (Table S1). Apparent post-rupture zones were mainly associated with actin bundles at the gap sides and often formed intercellular bridges corresponding to punctate adherens junctions^34^. The junction site of the bridges was associated with branched actin networks in 51.61 ± 50.8% of cases. PREM imaging also further verified that myosin motors co-localize with aligned actin bundles but not with branched actin networks at the junction (Figure S5d).

### A theoretical model for the three-way feedback between tension-dependent cadherin bond turnover, RhoA-associated actomyosin contractility, and Rac1-mediated actin polymerization

We propose a theoretical model to provide insight into the chemo-mechanical regulation of endothelial junctions. Combining our observations with experimental evidence from previous studies, we aim for our model to capture the following salient features of junctional remodeling:

(i) Force-sensitive cadherin bond formation and dissociation to govern cell-cell adhesion;
(ii) RhoA-dependent cytoskeletal contractility to strengthen or disrupt junctions;
(iii) Rac1-mediated actin polymerization to drive membrane protrusion.

#### Cadherin bond turnover

**Fig. 2:**
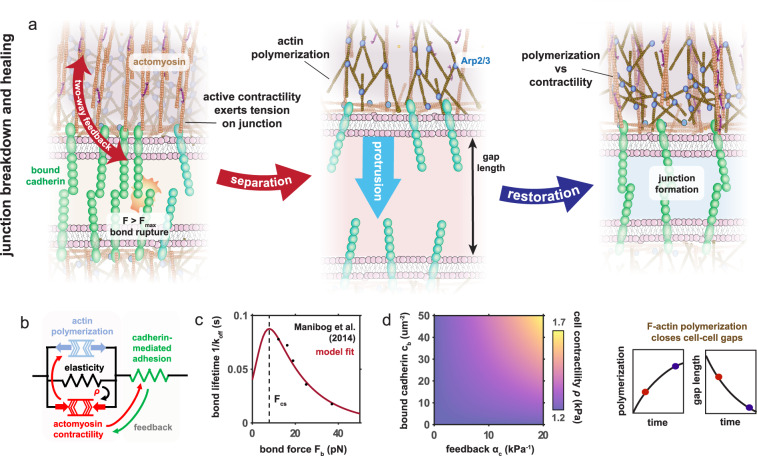
Feedback between mechanosensitive signaling and active forces governs endothelial junction remodeling. **a** Schematic highlighting the proposed model: In connected cells active cell tension stabilizes cadherin bonds which in turn anchor the actomyosin network and mediate signaling. High tension drives bond failure and cell separation (red). Following adhesion rupture Arp2/3 mediated actin polymerization generates protrusive stresses which can ultimately lead to junction restoration (blue); (**b**) chemo-mechanical model considers stresses induced by polymerization which are opposed by active and passive cell stresses. These parallel components act in series with the cadherin bonds which remodel in response to tension; (**c)** Experimental model calibration for the lifetime of a single VE-cadherin bond as a function of bond force. Cadherins present catch behavior under forces smaller than the catch-slip transition force ${F}_{{cs}}$, while they show slip behavior at forces larger than ${F}_{{cs}}$; (**d**) Predicted dependence of contractility $\rho$ on bound cadherin density ${c}_{b}$ and adhesion-mediated feedback strength ${\alpha }_{c}$.

The maintenance of vascular endothelial cell-cell junctions is predominantly governed by localization of VE-cadherin, which form homotypic bonds with corresponding proteins on the membrane of neighboring cells (Fig. 2a). Molecular-level studies indicate that cadherin bonds exhibit catch-bond behavior at low forces and slip-bond behavior at large forces^35–37^. We may consider the force within a cadherin bond to be given by ${F}_{b}={\kappa }_{b}{\Delta }_{b}$, where ${\kappa }_{b}$ is the effective cadherin stiffness and ${\Delta }_{b}$ its change in length. Following from the catch-slip model of Novikova and Storm (2013)^38^, the dissociation rate of a single bond can be described by

1 $${k}_{d}={k}_{0}\left({{\rm exp }}\left(\frac{{F}_{b}}{{F}_{s}}-{\phi }_{s}\right)+{{\rm exp }}\left({\phi }_{c}-\frac{{F}_{b}}{{F}_{c}}\right)\right),$$

where ${F}_{c}$ is the ‘catch’ force, ${F}_{s}$ is the ‘slip’ force, ${\phi }_{c}$ and ${\phi }_{s}$ are associated dimensionless catch-slip parameters, and ${k}_{0}=1{{s}}^{-1}$ is a reference dissociation rate scaled by the catch-slip response^38^. This model provides excellent agreement with experimentally measured lifetimes of single VE-cadherin bonds^36^ as shown in Fig. 2c, and naturally yields a catch-slip transition force (${F}_{{cs}}$), at which the bond lifetime reaches a maximum. When ${{F}_{b} < F}_{{cs}}$ the lifetime increases as the force increases, characteristic of ‘catch’ behavior; when ${F}_{b} > {F}_{{cs}}$ the lifetime decreases as the force increases, also referred to as the ‘slip’ regime. The binding probability ${P}_{b}$ for a VE-cadherin may then be expressed by $d{P}_{b}/{dt}=\left(1-{P}_{b}\right){k}_{a}-{P}_{b}{k}_{d}$, where the association rate ${k}_{a}$ is assumed to be constant. Considering a membrane with a sufficient VE-cadherin density ${c}_{{tot}}$ and assuming uniform load distribution at a adhered region^38^, the turnover in the density of bound cadherin ${c}_{b}$ can then be determined through a mean-field expression such that ${c}_{b}={P}_{b}{c}_{{tot}}$, whereby

2 $$\frac{d{c}_{b}}{{dt}}={k}_{a}{c}_{u}-{k}_{0}\left({{\rm exp }}\left(\frac{{F}_{b}}{{F}_{s}}-{\phi }_{s}\right)+{{\rm exp }}\left({\phi }_{c}-\frac{{F}_{b}}{{F}_{c}}\right)\right){c}_{b},$$

where ${c}_{u}$ is the unbound cadherin density and ${c}_{{tot}}={c}_{b}+{c}_{u}$. Further, we note a threshold force ${F}_{{crit}}$ for unbinding beyond which bonds will rupture, reported to be on the order of 22 ${pN}$^36^. Unbound cadherin may also be transported to and from the membrane through passive and active processes (e.g. endocytosis), further explored in SI Section S2.

#### Cytoskeletal contractility

Adherens junctions connect directly to force-generating actomyosin networks in the cell cytoplasm, which can apply tension to the cadherin bonds and increase their stability^29^. In turn, the formation of cadherin bonds at intercellular junctions triggers a range of signaling pathways that lead to the activation of RhoA using a variety of biochemical processes. For example, activation of the Src family of kinases (SFK)^39^ downstream of E-cadherin engagement leads to Src-mediated phosphorylation of Rho GDI, which reduces RhoA GDP sequestering and enables Rho GDP-GTP cycling via the activity of guanine nucleotide exchange factors (GEFs)^40,41^. Several RhoA GEFs have been implicated in RhoA activation at endothelial and epithelial adherens junctions, including p114 RhoGEF^42,43^, Ect2^44^, and Trio^45^, although the entire pathways of their activation are frequently unknown. Increased RhoA activates Rho-associated kinase (ROCK), which increases myosin activity via phosphorylation of the myosin light chain^46^ and thereby promotes higher cell tension via actomyosin contractility. Cytoskeletal tension also promotes the recruitment of vinculin to cell-cell junctions, which in turn mediates the recruitment of myosin motors^47^. We have previously developed a chemo-mechanical feedback model to describe the interdependence of stress ${\sigma }_{{ec}}$, signaling, and myosin-dependent contractile stress $\rho$^48^ (full derivation outlined in SI Section S1), extended here to consider explicit interactions with cell-cell adhesions such that

3 $$\frac{d\rho }{{dt}}=-{k}_{\rho }\left(\varepsilon \,+\beta \left(\rho -{\rho }_{0}\right)-{\sigma }_{{ec}}\left({\alpha }_{c}\left(\frac{{c}_{b}}{{\gamma }_{c}+{c}_{b}}\right)\,+{\alpha }_{0}\right)\,\right),$$

where $\varepsilon$ is the strain, ${\rho }_{0}$ is a relative myosin motor-generated contractile stress in the quiescent state, ${k}_{\rho }$ is a kinetic constant governing the rate of myosin recruitment, and $\beta$ denotes a chemomechanical coupling parameter regulating motor engagement. The $\beta \left(\rho -{\rho }_{0}\right)$ term ensures that cell contractility in the absence of stress ${\sigma }_{{ec}}$ is the quiescent value. ${\alpha }_{c}$ relates signaling feedback mediated by cadherin junctions (SFK, RhoA) where ${\gamma }_{c}$ denotes the cadherin concentration for half-strength signaling, and ${\alpha }_{0}$ relates to junction-independent pathways such as calcium influx through mechanosensitive channels, which downstream activates myosin light chain kinase (MLCK) and promotes cross-bridge cycling^49^. Thus, the ${\sigma }_{{ec}}\left({\alpha }_{c}\left(\frac{{c}_{b}}{{\gamma }_{c}+{c}_{b}}\right)+{\alpha }_{0}\right)$ term promotes cell contractility as governed by junction-associated signaling and cell stress (Fig. 2d). The stress that promotes myosin activity depends on both active and passive constituents, such that ${\sigma }_{{ec}}=\rho+{K}_{{ec}}\varepsilon$, where ${K}_{{ec}}$ is the effective passive cytoskeletal stiffness.

#### Actin polymerization-induced protrusive stress

In the event of adhesion failure, we observed that neighboring cells can reconnect by developing protrusions, mediated by actin polymerization at the cell periphery (Fig. 1). Another member of the Rho GTPase family, Rac1, activates the WAVE regulatory complex to drive Arp2/3-associated polymerization of branched actin networks (Fig. 2a)^50^. Compressive stress generated by such polymerization can drive protrusion of the cell membrane. As such, the effectors Rac1 and RhoA work in opposition, governing protrusion and contraction, respectively. Interestingly, Rac1 is also suppressed by RhoA activity; signaling through ROCK activates Rac1 GAPs, and myosin activation locally prevents recruitment of Rac1 GEFs^51^. We therefore propose a model to describe the stress generated by branched actin network polymerization ${\sigma }_{{{{\rm P}}}}$ such that

4 $$\frac{d{\sigma }_{{{{\rm P}}}}}{{dt}}={-k}_{{{{\rm P}}}}\left(1-\frac{{{{{{{{{\rm{\sigma }}}}}}}}}_{{{{\rm P}}}}}{{\sigma }_{{{{{\rm P}}}}_{0}}}-\frac{\rho }{{\gamma }_{\rho }+\rho }\right),$$

where ${\sigma }_{{{{{\rm P}}}}_{0}}$ is the maximum potential compressive stress induced by actin polymerization, associated with a maximum level of Rac1 signaling, and ${k}_{{{{\rm P}}}}$ is a kinetic constant. The first bracketed term describes the self-inhibition of Rac1 (and downstream polymerization) at high expression levels^52^, and the second term describes the antagonistic interactions between RhoA and Rac1 as determined by suppression constant ${\gamma }_{\rho }$; with increasing contractility $\rho$, Rac1-mediated actin polymerization will reduce. In our model we consider contractility $\rho$ as a proxy for RhoA signaling in relation to Rac1 inhibition. This approach could readily be integrated with explicit RhoA and Rac1 signaling dynamics in accordance with recent work^53^.

#### Model implementation

**Fig. 3:**
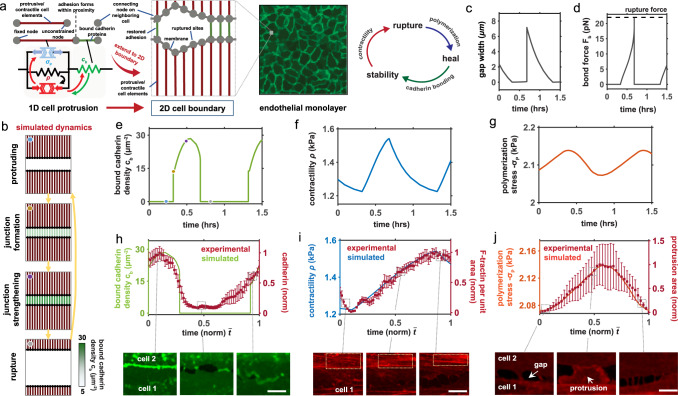
Dynamic junction rupture and recovery. **a** The 2D model discretely considers independent protrusions comprised of cytoskeletal (protruding/ contractile) elements (red), adhesion elements that can rupture and reform (green), and membrane/cortical actin elements that connect the protrusions (grey); (**b**) Simulated cell-cell boundary showing cycle of protrusion, junction formation, cadherin binding, and rupture at high tension; Predicted (**c**) gap size, (**d**) cadherin bond force, (**e**) bound cadherin density, (**f**) cell contractility, and (**g**) polymerization-induced stress (values are the same in all parallel elements). For all simulations ${\alpha }_{c}=20{kP}{a}^{-1}$ and ${\sigma }_{{{{{\rm P}}}}_{0}}=-\!3.725{kPa}$ unless otherwise stated; (**h**-**j**) Comparison of model predictions with experimentally measured cadherin ($n=6$ junctions), F-tractin ($n=4$ junctions), and protrusion area ($n=4$ junctions) with corresponding sample images of cell-cell boundaries (mean $\pm$ s.e.m.). Scale bars, 1 $\mu m$.

Initially, considering a cellular element of length ${L}_{{ec}}$ (Fig. 3a), in an open system (unconnected cells) stress equilibrium mandates that the strain in a linear cell element is given by $\varepsilon=-(\rho+{{{{{{{{\rm{\sigma }}}}}}}}}_{{{{\rm P}}}})/{K}_{{ec}}$. Therefore, within our model actin polymerization and actomyosin tension directly compete to enable cell protrusion or contraction (Fig. 2a) such that cell-cell can be restored if an inter-cell gap of reference length ${L}_{g}$ is sufficiently reduced (i.e. ${L}_{{ec}}\varepsilon \ge {L}_{g}$). Once the cells come into contact, bond formation and reinforcement can occur^1^ in accordance with Eq. 2, with the expression for stress equilibrium in a 1D system then given by:

5 $${\sigma }_{{tot}}=({L}_{g}{K}_{{ec}}+{L}_{{ec}}\left(\rho+{{{{{{{{\rm{\sigma }}}}}}}}}_{{{{\rm P}}}}\right))/({L}_{{ec}}+\frac{{K}_{{ec}}}{{\kappa }_{b}{c}_{b}}),$$

such that the force in an individual cadherin bond is then given by ${F}_{b}={\sigma }_{{tot}}/{c}_{b}$. A detailed derivation is provided in SI Section S3. Cadherin bonds will fail if the bond force exceeds a critical value ${F}_{{cr}{it}}$, returning to an open system. We proceed to extend our framework to the analysis of junction dynamics in an endothelial network. To explore junctional remodeling at a two-cell boundary, we develop a 2D spring lattice model assembled from the aforementioned 1D elements arranged in parallel and connected by membrane/cortical actin elements (Fig. 3a). Thus we discretely consider (i) independent protrusions comprised of active cytoskeletal (protrusive/contractile) elements, (ii) adhesion elements that can rupture, remodel, and reform, and (iii) passive membrane/cortical actin elements that connect the protrusions. Elements in the lattice are connected by nodes, and associated chemo-mechanical behavior within each element is governed by our proposed model (Eqs. 1–4). Note that we refer to a junction as the adhered boundary between two or more cells, and refer to adhesion at individual protrusion nodes in terms of the local density of cadherin bonds. The system is solved using a Newton-Raphson iterative scheme (see Methods) such that mechanical equilibrium is achieved at every node at every time point in a quasi-static analysis. Model parameter definitions and values are provided in Table S3 (SI Section S6) along with a sensitivity analysis of parameter influence on model predictions.

### Dynamic gap formation and recovery emerges from feedback between mechanosensitive signaling and active cytoskeletal forces

Having developed a model to describe how RhoA-driven contractility and Rac1-mediated actin polymerization interact to govern endothelial junction strength and recovery, we investigate how junctional remodeling depends on crosstalk between these processes. Initially considering a bicellular border, our model predicts that dynamic behavior emerges in cycles of cell protrusion, adhesion formation, junction strengthening, and rupture (Fig. 3b; Supplementary Movie 8). Polymerization-induced protrusive stresses reduce cell-cell gap width over time (Fig. 3c), ultimately facilitating intercellular contact, which enables spontaneous cadherin bond formation (Fig. 3e). This behavior can be directly observed in Supplementary Movies 1-2. As the density of bonds ${c}_{b}$ increases, the adhesion-dependent signaling feedback strength also increases (via Eq. 3), promoting an increase in cell contractility $\rho$ through myosin phosphorylation and crossbridge cycling (Fig. 3f). Within a cadherin bond catch regime, higher contractility reduces bond dissociation which overall promotes a higher computed bond density. To test these model predictions, we carried out time-series imaging of GFP-VE-cadherin and mScarlet-Myl9 (myosin light chain) at cell-cell interfaces, which revealed a concurrent increase in localized cadherin and myosin concentrations (Figure S6a-c; Supplementary Movie 12). In conjunction with increased cadherin density and contractility, our model suggests that polymerization-induced stress ${\sigma }_{{{{{{{{\rm{P}}}}}}}}}$ reduces, via RhoA-associated Rac1 suppression (Fig. 3g). Supporting our model predictions and observations, using high-speed imaging Yamada and Nelson (2007) observed that Arp2/3-induced lamellipodia rapidly give way to stress-activated RhoA recruitment following intercellular contact, driving a strengthening of nascent cell contacts^54^. Development of higher contractility in our model over time then pushes the adhesion into a slip-regime, driving rapid bond dissociation which in turn increases the force acting on individual bonds (Fig. 3d). Rupture ensues when this force exceeds the threshold value, causing cell-cell separation. Time-series analysis of remodeling at cell-cell junctions indeed revealed that further increases in myosin intensity was followed by a loss of local cadherin and subsequent junction failure (Figure S6a-c; Supplementary Movie 12). Kugelmann et al (2018) also demonstrated that increased RhoA-associated contractility following thrombin or histamine treatment causes a breakdown in adherens junctions^27^. In our model, a loss of adhesion-mediated signaling then lowers contractility (Fig. 3e-f), in turn facilitating a restoration of the polymerization-induced stress to reconnect cells (Fig. 3c,g). This process is predicted to cycle repeatedly, closely resembling the ‘stick-slip’ cycle often reported in mesenchymal-type migration^55,56^. We directly compared predicted junctional remodeling with our observations of gap formation and recovery at HUVEC boundaries (Fig. 3h–j) over normalized timescales, with the model showing excellent agreement with experimental observations. Gap formation corresponds with a reduction in cadherin density (Fig. 3h). In response to a gap forming, protrusions emerge from the cell edge (Fig. 3j) to re-establish cell adhesion. This is followed by an increase in cadherin density and cell contractility, with an associated decay in protrusion.

### Rupture primarily initiates at multi-cell junctions in an endothelial monolayer with healing mediated by recovery of actin polymerization

**Fig. 4:**
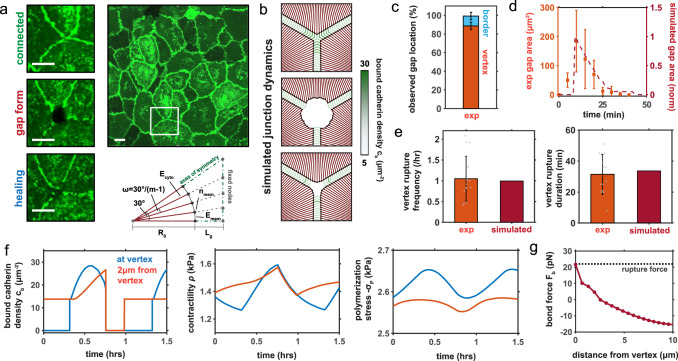
Simulations of Junction Dynamics at Cell Vertices. **a** Dynamic behavior of HUVECs expressing VE-cadherin-GFP cultured on a thin collagen gel, showing typical formation and healing of gaps at a multi-cell vertex. Inset frames recorded at 0 min, 70 min, and 130 min (top to bottom). Scale bar: ${{{{{{20}}}}}}\,{{{{{{\mu }}}}}}{{{{{{ m}}}}}}$; (**b**) Sample gap formation and healing within the simulated monolayer; (**c**) Experimentally observed gap locations in the HUVEC monolayer with rupture predominantly occurring at multicell vertices (mean ${{{{{{{\boldsymbol{\pm }}}}}}}}$ s.d., ${{{{{{ n}}}}}}{{{{{{=}}}}}}{{{{{{15}}}}}}$ gaps); (**d**) Measured and simulated endothelial gap area over time (mean ${{\pm }}$ s.d., ${{{{{{ n}}}}}}{{=}}{{{{{{15}}}}}}$ gaps); (**e**) Experimental and simulated rupture frequency at a vertex and duration of rupture (mean ${{\pm }}$ s.d., ${{{{{{n}}}}}}{{{{{{=}}}}}}{{{{{{15}}}}}}$ gaps); (**f**) Predicted polymerization, bound cadherin density, and contractility along the cell edge; (**g**) Predicted bond force along the adhered cell edge prior to rupture event indicating highest force is localized at the vertex. For all simulations ${{{{{{{\alpha }}}}}}}_{{{{{{{ c}}}}}}}{{{{{{=}}}}}}{{{{{{17}}}}}}{{{{{{kP}}}}}}{{{{{{{a}}}}}}}^{{{{{{{-}}}}}}{{{{{{1}}}}}}}$ and ${{{{{{{\sigma }}}}}}}_{{{{{{{{{P}}}}}}}}_{{{{{{{0}}}}}}}}{{=}}{{{{{{-}}}}}}{{{{{{4}}}}}}{{{{{{.}}}}}}{{{{{{725}}}}}}{{{{{{kPa}}}}}}$ unless otherwise stated.

With an understanding of the mechanism of junction failure and recovery, we were curious as to whether endothelial gap dynamics differed at bicellular junctions and multi-cellular vertices. We observed that gaps localized more frequently at multi-cellular junctions, growing and healing over time (Fig. 4a,d; Supplementary Movie 3). Quantitative measurements demonstrated that the probability of observing a gap at a vertex was approximately nine times higher than that at bicellular borders (Fig. 4c). We thus adapted our model geometry to consider a multi-cellular junction (Fig. 4a), as described in Methods. Our simulations and experimental analysis of multi-cellular junction behavior suggests that, when a gap forms, separation propagates along the boundary, which further increases the gap size (Fig. 4b,d; Supplementary Movie 9). Then, due to a loss in contractility (associated with adhesion rupture and signaling downregulation) and increasing polymerization, the cell membrane protrudes to reestablish contact and adhesion. Gaps were observed to form at the endothelial vertices approximately once per hour, with ruptures persisting over 30 minutes (Fig. 4e,f; Figure S7). Our simulated timescales provide excellent agreement with these data, further supporting the validity of our framework. Our analysis clearly indicates that cell activity is non-uniform along boundaries within a monolayer. Growing tension at vertex cadherin bonds causes them to enter a slip regime and subsequently the local adhesion fails (Fig. 4g). As the cell locally retracts in this region, additional stress is imposed on neighboring adhesions which causes the separation to propagate along the boundary. The rupture is arrested when the critical bond force within an adhesion is not surpassed and contact can thus be sustained. Over time, the adjacent membrane is predicted to protrude due to actin polymerization and loss of contractility, which enables cell contact and adhesion until the whole boundary is restored. With junctions restored along the cell-cell boundary, adhesions strengthen and contractility increases until rupture occurs again. Our model indicates that the forces exerted on cadherin bonds are significantly higher at vertices than at two-cell borders (Fig. 4e), which can drive more frequent bond rupture and adhesion failure; this emerges from a combination of constraint, alignment of the cortex and associated stress, and the high regional stretch required to develop contact at the vertex. To further assess the development of such vertex stress concentrations, we developed an analytical model which reveals that a stress singularity can arise at the multi-cellular junction when there is a mismatch between the cytoskeletal and adhesion stiffness. This analysis is discussed in detail within SI Section S5.

### Pharmacological treatments modulating cell contractility and actin polymerization drive weakening and failure of junctions

**Fig. 5:**
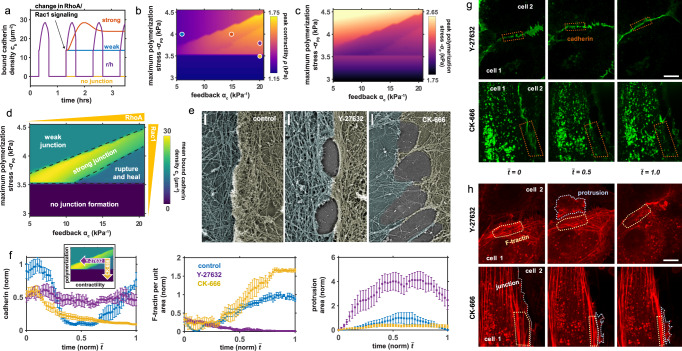
Influence of pharmacological treatments on junction stability. **a** Transients in bound cadherin following altered RhoA or Rac1 signaling which underpins strong, weak, rupture/heal junctions or disconnected cells. Corresponding parameters shown on (**b**); Predicted (**b**) cell contractility and (**c**) polymerization induced stress in the phase space spanned by RhoA-associated feedback parameter ${\alpha }_{c}$ and Rac1-associated maximum polymerization stress ${\sigma }_{P0}$; (**d**) Phase diagram of junction behavior as driven by actin polymerization and adhesion-mediated feedback, showing bound cadherin density; (**e**) PREM images of HUVECs showing cytoskeleton organization and junction state associated with control conditions and treatment with Y-27632 or CK666. Contacting cells are pseudo-colored to highlight cell boundary, which was identified based on immunogold VE-cadherin labeling. Scale bars, 0.5 $\mu m$; (**f**) Local intensity of cadherin ($n=6$ junctions), actin ($n=4$ (control), $n=6$ (Y27632), $n=5$ (CK666)), and protrusion area ($n=4$ (control), $n=4$ (Y27632), $n=5$ (CK666))) along cell-boundaries in response to Y-27632 or CK-666 treatment (mean $\pm$ s.e.m.); HUVEC adherens junctions showing (**g**) cadherin and (**h**) actin intensity over time in response to Y-27632 or CK-666 treatment. Scale bars, 1 $\mu m$.

Interestingly, our model predicts that a reduction in mechanosensitive signaling (via feedback parameter ${\alpha }_{c}$) or in the maximum polymerization-induced stress ${{{{{{{{\rm{\sigma }}}}}}}}}_{{{{{\rm P}}}}_{0}}$ changes junctional behavior (Fig. 5a) such that, beyond rupture and heal cycles, cells can form strong junctions, weak junctions, or lose the ability to recover their intercellular contact. Exploring the phase space of ${\alpha }_{c}$ and ${{{{{{{{\rm{\sigma }}}}}}}}}_{{{{{\rm P}}}}_{0}}$, analogous to the level of RhoA and Rac1 signaling, respectively, facilitates a mapping of computed cellular contractility (Fig. 5b), polymerization (Fig. 5c), and the junction state (Fig. 5d). With a critical balance of Rac1 and RhoA, a strong junction (high density of bound cadherin, ${c}_{b}$ along the cell-cell boundary) is predicted to develop due to the level of actomyosin force generation acting on the adhesions (Fig. 5b–d), which drives bond stabilization and reinforcement (via Eq. 2) in a catch-regime. This adhesion-stabilizing influence of cell contractility has not been widely appreciated, but recent studies now indicate that RhoA can induce junction contraction and strengthening^22,23^. Junctions can be further reinforced through vinculin unfolding and protein recruitment^57^; this latter pathway is explored in SI Section S2. A reduction in RhoA (via ${\alpha }_{c}$) is predicted to weaken cell-cell adhesion, in the form of intercellular contact but an absence of bond tension and stability. The low corresponding cell contractility fails to promote catch bond formation and reinforcement, further mitigated by a high levels of Arp2/3-mediated actin polymerization and associated compressive stress (Fig. 5b,c). To test these predictions, we treated the HUVEC monolayer with Y-27632, a Rho-kinase (ROCK) inhibitor. Observation of the junction and cytoskeleton microstructure by PREM revealed an absence of aligned actomyosin bundles, as anticipated, and dense branched actin structures indicative of Arp2/3-mediated actin polymerization (Fig. 5e). Gaps were apparent at cell boundaries, suggestive of weak adhesion. This is further supported by live-cell imaging at the cell-cell interface (Fig. 5f-g; Supplementary Movies 4-5); bound cadherin densities were lower in cells treated with Y-27632 than in untreated (control) cells, in agreement with model predictions. Cell contractility also reduced with treatment, demonstrated by a loss of dense actomyosin bundles which corresponded with an amplification of surface protrusions driven by actin polymerization (Fig. 5f,h). Our simulations suggest that impairment of Arp2/3-mediated polymerization (low ${{{{{{{{\rm{\sigma }}}}}}}}}_{{{{{\rm P}}}}_{0}}$) prevents gap closure and bond formation (Fig. 5d), as generated compressive stresses are insufficient to drive the development of protrusions. Indeed, treatment of HUVECS with CK-666, an Arp2/3-complex inhibitor, led to a loss of branched actin structures and surface protrusions (Fig. 5e) and the formation of large irrecoverable gaps between cells (Fig. 5f–h; Supplementary Movies 6-7). Our model also indicates that high level of Rac1 signaling (via ${{{{{{{{\rm{\sigma }}}}}}}}}_{{{{{\rm P}}}}_{0}}$) and associated compression of the cell-cell boundary may prevent the transmission of tensile forces through cadherin bonds and therefore impair adhesion integrity and cell contractility (Fig. 5d). In support of this prediction, Knezevic et al. (2009) report that platelet-activating factor, an activator of the Rac1 pathway, drives junction breakdown as characterized by cadherin endocytosis^33^.

**Fig. 6:**
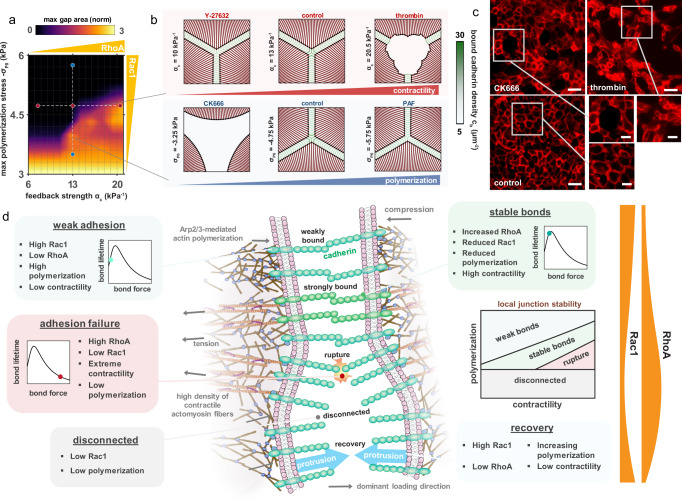
Junction state is governed by RhoA and Rac1 signaling. **a** Maximum predicted gap size as a function of feedback strength ${{{{{{{\alpha }}}}}}}_{{{{{{{c}}}}}}}$ and maximum polymerization-induced stress ${{{{{{{\sigma }}}}}}}_{{{{{{{{{ P}}}}}}}}_{{{{{{{{\bf{0}}}}}}}}}}$ (area normalized by maximum area under control conditions); (**b**) Predicted influence of pharmacological treatments on junction stability and gap formation. Scale bar, 25 ${{{{{{\mu }}}}}}{{{{{{m}}}}}}$; (**c**) Organization of HUVEC monolayer in response to pharmacological treatment with thrombin or CK-666. Scale bar, 50 ${{{{{{\mu }}}}}}{{{{{{m}}}}}}$. Red fluorescence shows CD31. Inset scale bar, 25 ${{{{{{\mu }}}}}}{{{{{{m}}}}}}$; (**d**) A balance of RhoA and Rac1 signaling is essential to the maintenance of endothelial junctions with variations driving junction weakening or failure.

We further investigate the role of Rac1 and RhoA signaling on the maximum gap size that emerges in the monolayer (Fig. 6a); simulations suggest that increasing Arp2/3-mediated polymerization can reduce gap formation and limit rupture propagation, while high signaling feedback to myosin phosphorylation and contractility drives larger gaps and further separation. In agreement with our findings, angiopoietin, a Rac1 activator and RhoA inhibitor^58^, has been shown to significantly reduce both endothelial gap size and rupture propagation^59^. We can again relate specific levels of RhoA and Rac1 expression to cell behavior in response to associated pharmacological treatments. At a critical balance between these signals, stable adhesion maintained at steady state (Fig. 6b-c). The region of highest bound cadherin density is predicted to emerge at the vertex. Our model suggests that further activation of RhoA and downstream actomyosin contractility will drive increased gap formation and junction breakdown (Supplementary Movie 10). In support of these findings, we found that treatment with thrombin, an activator of the RhoA signaling pathway, increases junction permeability (Fig. 6c; Figure S8b). Conversely, a reduction in cell contractility is predicted to reduce the cadherin bond density and thus weaken cell-cell adhesion (Fig. 6b). Indeed, we observed that the density of gaps increased following treatment with Y-27632 (Figure S8d). Walsh et al. (2001) also demonstrated that limiting ROCK activation is detrimental to junction integrity^31^. RhoA and Rac1 have opposing effects on cell protrusion, and as such simulations indicate that upregulation of Rac1 (e.g. through endothelial cell treatment with platelet-activating factor (PAF)), also causes junction weakening due to a reduction in VE-cadherin bond tension (Fig. 6b). Although we did not observe significant differences in gap areas following PAF treatment (Figure S8e), cells were visibly more compacted. Moreover, in agreement with model predictions, PAF has previously been shown to cause barrier disruption and cadherin endocytosis^33^, with endocytosis reported to occur under low junction tension^60^. Our model also suggests that insufficient actin polymerization can inhibit junction formation, as cells cannot sufficiently bridge the gap between their neighbors. Abu Taha and Schnittler (2014) also report that Rac1 mediates the bridging of intercellular gaps and formation of stable junction contacts^1^. Further analysis of the HUVEC monolayer reveals that large gaps emerge following blockage of Arp2/3 and associated actin branching through treatment with CK-666 (Fig. 6c; Figure S8c). Interestingly, we observed that treatment with thrombin or CK-666 led to the formation of large gaps that propagated along several cells (Figure S8f), whereas following treatment with Y-27632 or PAF gaps that formed were typically smaller than the neighboring cell. In summary, our model indicates that both RhoA and Rac1 expression have a bimodal influence on junction integrity, which should be appreciated when drawing conclusions from pharmacological regulation. Further, a critical balance between these signaling processes may be required for the maintenance of stable adhesion (Fig. 5d; Fig. 6d).

## Discussion

In this study, we propose a chemo-mechanical model to investigate how endothelial gaps are regulated within the microvasculature. Junction breakdown and recovery depend on the three-way feedback between active force generation, cadherin bond turnover, and actin polymerization. Assembly and disassembly of cadherin bonds at the cell-cell interface is force-dependent, with cadherin proteins exhibiting catch-bond behavior at low forces and slip-bond behavior at high forces^36^. In connected cells, actomyosin contractility is therefore shown to significantly affect adhesion stability, such that actively generated tension can increase bond lifetime but excessive stress causes bond dissociation and adhesion rupture (Figs. 5d, 6d and S6). The recruitment of myosin motors is a mechanosensitive process that occurs downstream of signaling from junction tension. Thus, a feedback loop emerges between adhesion stability and actomyosin contractility, whereby the formation and stretching of cadherin bonds upregulates signaling (via coupling parameter ${\alpha }_{c}$) to promote myosin-mediated force generation, in turn transmitting higher tension to the junction. Following the formation of endothelial gaps, Rac1-associated actin polymerization can aid adhesion recovery by protruding the membrane to facilitate cell-cell contact; this process is further advanced through the reduction in cell contractility associated with loss of adherens junctions and related signaling. Our model predicts that periodic gap formation emerges in cycles of cell protrusion, adhesion formation, junction strengthening, and rupture. We also observed such repeated gap formation in our experiments (Supplementary Movies 1 and 11). However, it is worth noting that such in-vitro remodeling is not necessarily periodic, due to the inherent stochastic nature of the biological activity, which is explored through a model extension in SI Section S8. Our model and experiments indicate that insufficient Rac1 signaling (implicitly considered through ${{{{{{{{\rm{\sigma }}}}}}}}}_{{{{{\rm P}}}}_{0}}$) may prevent gap closure in agreement with our previous findings^30^, while high Rac1 expression can lead to weak junctions characterized by a low bound cadherin density. The work of Knezevic et al. (2009) supports this latter result, who observed that high activation of Rac1 drives junction weakening^33^. Our simulations suggest that a balance between RhoA and Rac1 signaling is required to maintain a stable junction state (Figs. 5–6); reduced RhoA or Rac1 expression led to weak adhesions or perforated cell-cell contacts. These results were supported by live-cell imaging and pharmacological treatment of cultured HUVEC monolayers (Fig. 5e–h). Simulations also suggest that high levels of RhoA expression and associated actomyosin contractility drive adhesion failure; in agreement, treatment with thrombin drove a disassembly of endothelial junctions (Fig. 6c). Thrombin induces a rapid activation of RhoA and myosin contractility in endothelial cells^61,62^. The model proposed in this study can therefore help to resolve the seemingly contradictory effects reported for RhoA and Rac1 expression, by which through coupled interactions they can drive junction assembly, failure, recovery, or stability as dependent on their associated levels and crosstalk.

We also explored how spatial variations in gap formation can emerge at multi-cellular vertices within an endothelial monolayer. We observed the propagation of gaps along cell-cell boundaries and subsequent healing in a monolayer of HUVECs, in agreement with the timescales predicted by our model. Gaps had a tendency to form more frequently at multi-cellular junctions than at two-cell borders. Our numerical simulations suggest that the forces exerted on cadherin bonds are significantly higher at vertices than at bicellular boundaries, which can drive bond rupture and loss of adhesion, thereby promoting increased adhesion failure at multi-cellular junctions. Further, in SI Section S5 we show analytically that at a vertex, stress concentrations can arise from a mismatch between adhesion and cytoskeletal stiffness. The magnitude of the stress singularity at the triple-cell junction increases as the stiffness mismatch increases. Our numerical analysis also predicts the impact of pharmacological modulation of signaling and downstream cytoskeletal remodeling, with larger gaps predicted to form with high RhoA expression or low levels of actin polymerization. We experimentally tested predictions by treating HUVECs with thrombin, Y-27632, CK-666, and PAF (Fig. 6c-d; SI Figure S8) which showed good agreement with simulated behavior. Future studies could further test our model by exploring Src-specific inhibition and consequent changes to actomyosin contractility and gap formation^63^. Our vertex model assumes hexagonal symmetry, which greatly simplifies the problem as a representative element of the system need only consist of one-twelfth of a cell. This assumption facilitates direct investigation of gap localisation and adhesion complex rupture forces without the secondary complexity of asymmetric junction formation, while also significantly reducing simulation time. However, the model could readily be extended to explore the influence of asymmetric cell boundaries or direct mapping to experimental monolayer configurations.

Adherens junctions play a critical role in supporting many physiological processes, including collective cell migration^64^ and morphogenesis^65^. Clearly, such biological systems could be analyzed using our framework to provide mechanistic insight into their dependence on the crosstalk between active contractility and actin polymerization. Future advancements should also focus on detailing the feedback between integrin signaling and cadherin bond reinforcement^66^, building on our previous work exploring the interdependence of focal adhesion development and cell force generation^67,68^. The role of cell-cell interactions in endothelium homeostasis could also be investigated, to determine the influence of region-specific loading in the remodeling of vascular and myocardial endothelium. Notably, Manibog et al (2014) observed that the peak of the catch-slip curve is attained at lower forces with reduced calcium concentrations; future work could explore the feedback between mechanosensitive ion dynamics^69^, cadherin bonding, and cell contractility. Further, in our main analysis we limit ourselves to the consideration of a constant total cadherin density at each local adhesion site; however, the influence of active transport processes (endo- and exocytosis) on adhesion strength is explored in SI Section S3, with our analysis suggesting that force-mediated signaling can have a secondary indirect effect on bond stability by driving cadherin reinforcement through reduced endocytosis. Interestingly, Cavanaugh et al (2022) recently reported that activated RhoA can stimulate cadherin recruitment in the absence of visible junction contraction^23^. Future implementations of our model could explore this effect, to further study the competition between evolving tension and cadherin reinforcement.

The activity of the Rho family of GTPases in the endothelium has major connotations for vascular behavior. Vascular barrier function can be compromised during inflammation, with the inflammatory mediator thrombin driving endothelial hyper-permeability through RhoA activation^25,70^, thereby promoting extravasation of blood constituents. Our analysis suggests that this occurs through increased actomyosin contractility pushing cadherin bonds into a slip regime and subsequent rupture. Previous experiments have also identified that Rac1 suppression alone is sufficient to disassemble endothelial junctions^71^. Our simulations indicate that this effect is two-fold: reduced protrusion from a loss of actin polymerization lowers the compressive stress acting on the junction, thereby increasing bond tension and dissociation, and sufficient protrusive forces cannot then be generated to reconnect the cells. The formation of atherosclerotic lesions has been linked to endothelial dysfunction^72^, with such dysfunction also dependent on the RhoA/ROCK pathway^73^ and cell contractility. Conversely, pharmacological inhibition of Rac1 has been reported to rescue endothelial dysfunction^74^. As plaque development initiates early in life^75^, increased preventative measures should be supported to reduce the burden; future advancements could see our model extended to predict endothelial dysfunction and to develop a computational tool to advise on patient risk and early diagnosis of atherosclerosis. Beyond vascular disease, endothelial junction dynamics are also critical to cancer progression and metastasis. Tumor cells have been observed to exploit disrupted connectivity at multi-cellular junctions to transmigrate through the endothelium^76^. Prior work has considered endothelial gap formation^77^ by modeling constant contractile and protrusive forces that were randomly activated or deactivated over time, which provides an understanding of how junctions could break down to facilitate cancer cell extravasation. While the biochemical or biophysical signals that governed this process were unclear, there appeared to be a mechanism by which the tumor cells could identify vertices to increase their extravasation potential. The current work entails a significant advance over prior analyses, by integrating the dynamic remodeling of actomyosin networks and cell-cell adhesions with signaling feedback governed by cell mechanosensation. Simulations suggest that gap formation is critically dependent on adhesion-mediated signaling that upregulates actomyosin contractility, with junction restoration driven by a subsequent recovery in actin polymerization. We further highlight a mechanism by which junctions are most likely to break down at multi-cellular interfaces; cell constraint drives higher actomyosin tension and the emergence of concentrated adhesion stress in these regions. In summary, our findings suggest that the three-way feedback between actomyosin contractility, VE-cadherin bond turnover, and actin polymerization governs the regulation of endothelial gaps. Our proposed model can help guide the development of therapeutics that target the Rho family of GTPases and downstream active mechanical processes.

## Methods

### Live cell imaging of endothelial junctions

HUVECs (Lonza, CC-2519) were cultured in Endothelial Cell Basal Medium (Lonza, CC-3121) with supplements (Lonza, CC-4133) and maintained for no longer than six passages. For PREM experiments, HUVECs were plated on coverslips coated with ∼50 μg/ml (5 mg/cm^2^) collagen from rat tail (BD Biosciences, 354236). Y-27632 (Y100500; Toronto Research Chemicals) was prepared from 10-mM stock in water and added to culture medium to a final concentration of 50 μM for 1 h. CK666 (SML0006; Sigma-Aldrich) was prepared from 10-mM stock in DMSO and used at 100 μM for 40–60 min. For live-cell imaging, cells were plated on collagen-coated glass-bottomed dishes (MatTek Corporation) and maintained during imaging at 37 °C in humidified atmosphere with 5% CO_2_ using an UNO stage-top incubator (Okolab) with 20–30 min allowed for cell accommodation before imaging. Time-lapse sequences were acquired every 1 min for 60–70 min. Vehicle (DMSO for CK666 and water for Y-27632) or drugs were added during a pause between the ninth and 10th frames at the same concentration as for experiments with fixed cells. Imaging was performed using an Eclipse T*i* inverted microscope (Nikon) equipped with a CSUX1 spinning disk (Yokogawa Electric Corporation), CFI60 Plan Apochromat Lambda 20× 0.75-NA and CFI60 Apochromat total internal reflection fluorescence 100× 1.49-NA oil immersion objectives, and a QuantEM 512SC digital camera (Photometrics) driven by NIS Elements software (Nikon). Multiple positioning in the x–y plane and single-slice spinning-disk confocal mode with 488- and 561-nm laser wavelengths and FITC and TRITC filters were used for imaging. FIJI software (ImageJ; National Institutes of Health) was used to adjust image contrast and create montages. To measure cadherin concentration, we defined a region of interest (ROI) near the gap using the freehand selections tool in ImageJ. The width of the ROI was similar to the width of the bright area and its length was equal to maximum length of the opening. Then the mean grey value was recorded at the ROI for each time step. To quantify actin, we defined a ROI in the vicinity of the gap location using the polygon sections tool in ImageJ which encompassed actin fibers (F-tractin or LifeAct). We noticed that an increase in the level of contractile force in the cell was accompanied with an increase in intensity of the actin fibers, while the size of the ROI containing the fibers shrank. Therefore, to normalise the data we divided the intensity value from the ROI to the area of the ROI for each time step. To measure myosin (mScarlet Myl9) concentration, we defined a region of interest (ROI) near the junction using the freehand selections tool in ImageJ. Then the mean grey value was recorded at the ROI for each time step. pLV_iRFP_P2AT2A_mScarlet-I_Myl9^78^ was a gift from Sean R Collins (Addgene plasmid # 172474; http://n2t.net/addgene:172474; RRID:Addgene_172474). To find the protrusion area, an ROI encompassing the protrusion was defined using the freehand selections tool in ImageJ and the area of the protrusion was recorded at each time step. All measurements were carried out on at least four junctions from three independent experiments.

### Platinum-replica electron microscopy (PREM)

Following our previously developed protocol^79^, HUVEC monolayers were extracted with 1% Triton X-100 in PEM buffer (100 mM PIPES-KOH, pH 6.9, 1 mM MgCl_2_, and 1 mM EGTA) containing 2% polyethelene glycol (mol. wt. 35,000), 5 µM phalloidin and 2 µM taxol for 3 min, washed with PEM, incubated for 30 min with a primary mouse monoclonal antibody for VE-cadherin (cadherin-5, clone 75, BD Biosciences, #610251, 1:20) and a primary rabbit antibody for nonmuscle myosin IIA (#BT-567, Biomedical Technologies, 1:20) in the PEM buffer containing 5 µM unlabeled phalloidin and 2 µM taxol, washed with the PEM buffer and fixed with 0.2% glutaraldehyde in 0.1 M Na-cacodylate, pH 7.3. After quenching with 2 mg/ml NaBH_4_ in PBS for 10 min cells were blocked with 1% BSA in buffer A (20 mM Tris-HCl (pH 8), 0.5 M NaCl, 0.05% Tween 20), stained with a secondary anti-mouse IgG antibody conjugated to 18 nm colloidal gold (Jackson ImmunoResearch Laboratories, #115-215-166, 1:5) and anti-rabbit IgG antibody conjugated to 12 nm colloidal gold (Jackson ImmunoResearch Laboratories, #711-205-152, 1:5) and postfixed with 2% glutaraldehyde in 0.1 M Na-cacodylate, pH 7.3. Fixed cells were sequentially treated with 0.1% tannic acid and 0.2% uranyl acetate in water, critical point dried, coated with platinum and carbon, and transferred onto electron microscopic grids for observation. PREM samples were analyzed using JEM 1011 transmission electron microscope (JEOL USA, Peabody, MA) operated at 100 kV. Images were captured by ORIUS 832.10 W CCD camera (Gatan, Warrendale, PA) and presented in inverted contrast. Pseudocolors were applied using Hue/Saturation tool in Adobe Photoshop to avoid obscuring structural details. For treatment cases, Y-27632 (Y100500; Toronto Research Chemicals) was prepared from 10-mM stock in water and cells were first treated with $50{{{{{{{\rm{\mu }}}}}}}}{{{{{{{\rm{M}}}}}}}}$ Y-27632 in culture medium for 1-hr. CK666 (SML0006; Sigma-Aldrich) was prepared from 10-mM stock in DMSO and used at a concentration of 100 $\mu {{{{{{{\rm{M}}}}}}}}$ for 40–60 min.

### Immunofluorescence microscopy

Immunofluorescence staining of HUVECs was performed after simultaneous fixation and extraction with the mixture of 4% formaldehyde (#15710; Electron Microscopy Sciences) and 0.3% Triton X-100 in PBS for 15 min. After washing with PEM buffer, extracted unfixed cells were incubated with the primary antibody, washed with the PEM buffer, and fixed with 0.2% glutaraldehyde. After quenching with 2 mg/ml NaBH4 in PBS for 10 min, cells were stained with Alexa Fluor 488 phalloidin (Invitrogen, # A12379, 1:200) and Alexa Fluor 594 anti-rabbit IgG antibody (Fisher Scientific, # A11037, 1:100). Coverslips were mounted to slides with ProLong Gold antifade mountant (#P36941; Molecular Probes). Light microscopy was performed using an Eclipse TE2000-U inverted microscope (Nikon) equipped with Plan Apochromat 20× 0.75-NA and Apochromat 100× 1.3-NA objectives and Cascade 512B CCD camera (Photometrics) driven by MetaMorph imaging software (Molecular Devices).

### Vertex dynamics in monolayers

To mimic endothelial monolayer in a physiological state and enable high-resolution confocal live-imaging of endothelial gaps, HUVEC monolayers were cultured on top of thin layers (~60 µm) of collagen. Briefly, HUVECs (Lonza, expanded to passage 3) were transduced with GFP tagged VE-cadherin^80^ and expanded and cryopreserved at passage 6. Transduced HUVECs (350,000 at passage 7) were seeded on top of collagen substrate (rat tail type I, Corning, 2.5 mg/ml, thickness of ~60 µm) formed in the central region of Mattek glass-bottom dish prior to seeding. Two days following seeding, a uniform semi-permeable HUVEC monolayer with physiologically relevant permeability (tested with dextran diffusion) was formed. The dynamics of gap opening were captured over 3 hrs (every 10 min) using live confocal imaging (Olympus FV1000, 63X oil objective) to image the dynamics of VE-cadherin under physiological incubation condition (37 °C, 5% CO2). To improve the quality of imaging, in a few cases Alexa Fluor 647-conjugated PECAM-1 antibody (Hu CD31, BD BioSciences) was applied to the monolayer and washed to image junctional dynamics for a short period of time (~1 hr). To obtain enough measurements for statistical analysis, six regions on three separate dishes containing fully confluent HUVEC monolayer were experimentally tested. Fluorescence time-lapse images of cell junctions were manually analyzed using ImageJ to measure frequency and duration of gaps identified as void regions along junctions with sizes greater than ~2 µm. Considering that the monolayer thickness was as thin as 3-4 um (resting endothelial cells), we used a maximum intensity method in ImageJ to apply a Z-projection to the image stacks. This provided us a clear picture of the position of the gaps and enabled us to quantify the area of the gap over time. Then the rupture duration was calculated for seven gaps from four independent samples. For pharmacological perturbation, HUVECs were plated at 4200 cells/mm2. After 24 hours, cells were treated with either thrombin (0.01 U/mL; Millipore-Sigma, SKU: 10602400001), CK-666 (200 nM; EMD Millipore, 182515), PAF (100 nM; Fisher Scientific Tocris, 29401), Y-27632 (30uM; EMD Millipore, 5092280001), or left untreated overnight. Next day, wells were washed once with DPBS, then incubated for 10 minutes with Alexa Fluor 647 Mouse Anti-Human CD31 (BD Bioscience, 561654) at 1:20 dilutions in EGM-2 media. Cells were rinsed twice with EGM-2 media, then imaged by fluorescent microscopy using a Nikon Eclipse Ti-2E microscope. To measure gap areas, we defined regions of interest (ROIs) around gaps using the freehand selections tool in ImageJ and computed the encompassed area in a minimum of five images for each treatment case.

### Cell network dynamics model development

Cells are assumed to form repeating hexagonal shapes in a monolayer when spread and adhered (Fig. 4a). Taking advantage of the symmetry in this system, we model one-twelfth of an initially-circular cell body. The cell is discretized into nodes, connected by a system of elements that describe the chemo-mechanical behavior of the cytoskeleton, membrane/cortical actin, and cell-cell adhesions (Fig. 4). Considering a given element ${E}_{{ij}}$ connected to nodes ${{{{{{{{\boldsymbol{n}}}}}}}}}_{i}$ and ${{{{{{{{\boldsymbol{n}}}}}}}}}_{j}$, its length ${L}_{{Eij}}$ may be determined by computing the current distance between the nodes, with ${L}_{{Eij}}={||}{{{{{{{{\boldsymbol{n}}}}}}}}}_{i}-{{{{{{{{\boldsymbol{n}}}}}}}}}_{j}{||}$. With a reference length ${L}_{{Eij}}^{0}$, the force vector associated with element deformation may then be determined by ${{{{{{{{\boldsymbol{F}}}}}}}}}_{{ij}}={\kappa }_{{ij}}\left({L}_{{Eij}}-{L}_{{Eij}}^{0}\right)\frac{{{{{{{{{\boldsymbol{n}}}}}}}}}_{j}-{{{{{{{{\boldsymbol{n}}}}}}}}}_{i}}{{L}_{{Eij}}}$, where ${\kappa }_{{ij}}$ is the spring stiffness of the element in units $N/\mu m$. Element forces act on the connected nodes, such that the total force vector for a given node ${{{{{{{{\boldsymbol{n}}}}}}}}}_{i}$ is described by ${{{{{{{{\boldsymbol{F}}}}}}}}}_{i}=\mathop{\sum}\limits_{j\in {neighbor}(i)}{{{{{{{{\boldsymbol{F}}}}}}}}}_{{ij}}$, where $j$ iterates through all nodes that share an element with node ${{{{{{{{\boldsymbol{n}}}}}}}}}_{i}$. We then solve our nodal system for mechanical equilibrium such that the total force acting on every node is zero (i.e. ${{{{{{{\boldsymbol{F}}}}}}}}\left({{{{{{{\boldsymbol{n}}}}}}}}\right){{{{{{{\boldsymbol{=}}}}}}}}0$). In keeping with previous methodologies we assume that inertial forces have no significant impact on the system^77^. Due to the non-linearity of the equations, we use a Newton-Raphson numerical scheme to attain a solution^81^.

For vertex simulations we consider a system of $m$ cytoskeletal elements ${{{{{{{{\boldsymbol{E}}}}}}}}}_{{cyto}}$ connected to a fixed node at the cell center (Fig. 4a); in this study $m=21$. The elements have a reference length equal to the initial radius of the cell ${R}_{0}$ and are aligned in the radial direction, equally separated at an angle $\omega=30^\circ /(m-1)$. It should be noted that ${\sigma }_{{ij}}$ and ${\varepsilon }_{{ij}}$ refer to the stress and strain within an element ${E}_{{ij}}$ connected by nodes $i$ and $j$, with the element strain given by ${\varepsilon }_{{ij}}=1-{L}_{{Eij}}/{L}_{{Eij}}^{0}$. Contractility ${\rho }_{{ij}}$ and polymerization-induced stress ${\sigma }_{{{{\rm P}}},{ij}}$ are modeled as described by Eqs. 3 and 4. The stress in a cytoskeletal element (${E}_{{ij}}\in {{{{{{{{\boldsymbol{E}}}}}}}}}_{{cyto}}$) is given by ${\sigma }_{{ij}}={\rho }_{ij}+{\sigma }_{{{{\rm P}}},{ij}}+{K}_{{ec}}{\varepsilon }_{{ij}}$ and the stress in an adhesion element (${E}_{{ij}}\in {{{{{{{{\boldsymbol{E}}}}}}}}}_{{adh}}$) ${\sigma }_{{ij}}={\varepsilon }_{{ij}}{K}_{J}$. Here, ${K}_{J}={k}_{b}{c}_{{bij}}{L}_{{ij}}^{0}$ is the effective adhesion element stiffness. ${\sigma }_{{{{\rm P}}},{ij}}$ is not considered within the stress term in Eq. 3, as it would incorrectly cause a reduction in contractility when a junction forms. The passive cytoskeletal stiffness ${K}_{{ec}}$ may be converted to a spring stiffness via ${\kappa }_{{ec}}={K}_{{ec}}{A}_{0}/{R}_{0}$, where ${A}_{0}$ is a reference cross-sectional area assumed to be constant for all elements. This force acts in parallel with the active cytoskeletal force. At any given time point, we can compute the active force such that ${F}_{{ij}}^{a}=({\rho }_{{ij}}+{\sigma }_{{{{\rm P}}},{ij}}){A}_{0}$, with the total cytoskeletal element force given by ${{{{{{{{\boldsymbol{F}}}}}}}}}_{{ij}}=({\kappa }_{{ec}}({L}_{{Eij}}-{L}_{{Eij}}^{0})+{F}_{{ij}}^{a})\frac{{{{{{{{{\boldsymbol{n}}}}}}}}}_{j}-{{{{{{{{\boldsymbol{n}}}}}}}}}_{i}}{{L}_{{Eij}}}$, if ${E}_{{ij}}\in {{{{{{{{\boldsymbol{E}}}}}}}}}_{{cyto}}$. The outer cytoskeletal nodes are connected by membrane elements ${{{{{{{{\boldsymbol{E}}}}}}}}}_{{mem}}$, whose passive forces are similarly described by ${{{{{{{{\boldsymbol{F}}}}}}}}}_{{ij}}={\kappa }_{{mem}}({L}_{{Eij}}-{L}_{{Eij}}^{0})\frac{{{{{{{{{\boldsymbol{n}}}}}}}}}_{j}-{{{{{{{{\boldsymbol{n}}}}}}}}}_{i}}{{L}_{{Eij}}}$, if ${E}_{{ij}}\in {{{{{{{{\boldsymbol{E}}}}}}}}}_{{mem}}$. Cytoskeletal elements are constrained to displace only in the radial direction (${u}_{\theta }=0$). Adhesions form and fail between nodes on membrane and those on the neighboring cell. We consider that the membrane node at the end of each protrusion has a surface perpendicular to the orientation of the protrusion with a total cadherin density ${c}_{{tot}}$ and area ${A}_{0}$. When the protrusion node contacts a neighbouring cell, cadherin bonds may form and turnover such that there is an evolving density of bound cadherin ${c}_{b}$ and unbound cadherin ${c}_{u}$. To describe this activity, we again take advantage of symmetry and consider there to be a number of fixed nodes along the hexagonal boundary with which a cell-cell adhesion element ${{{{{{{{\boldsymbol{E}}}}}}}}}_{{adh}}$ may form (described in more detail in the next section). The spring stiffness of these adhesion elements is given by ${\kappa }_{J,{ij}}={\kappa }_{b}{c}_{b,{ij}}{A}_{0}$, such that the associated element force is ${{{{{{{{\boldsymbol{F}}}}}}}}}_{{ij}}={\kappa }_{J,{ij}}({L}_{{Eij}}-{L}_{{Eij}}^{0})\frac{{{{{{{{{\boldsymbol{n}}}}}}}}}_{j}-{{{{{{{{\boldsymbol{n}}}}}}}}}_{i}}{{L}_{{Eij}}}$, if ${E}_{{ij}}\in {{{{{{{{\boldsymbol{E}}}}}}}}}_{{adh}}$. It should be noted that when the membrane nodes are disconnected the element stiffness tends to zero (i.e. ${\kappa }_{J,{ij}}\to 0$).

### Simulation procedure

The model is implemented using an ODE solver (ode15s) in Matlab (R2020b, Mathworks). An event function is defined in the option structure of ode15s to identify (i) when a node on the cell membrane is within contact distance of a corresponding node on a neighboring cell membrane, or (ii) when the force in cadherin bonds exceeds a critical rupture force. This event respectively triggers the formation of adhesive bonds between the cells described by an element with stiffness ${\kappa }_{J,{ij}}={\kappa }_{b}{c}_{b,{ij}}{A}_{0}$ and reference length ${L}_{J}$, or adhesion failure via the element stiffness tending to zero (${\kappa }_{J,{ij}}\to 0$). The simulation proceeds as follows:

1. Initialize a symmetric cell of radius ${R}_{0}$ with minimum gap distance ${L}_{g}$ from its neighbor (Fig. 4a).
2. Solve the network force balance and evolution of cytoskeletal dynamics over time.
3. At every time point assess if the position of any membrane node ${{{{{{{{\boldsymbol{n}}}}}}}}}_{i}\in {{{{{{{{\boldsymbol{n}}}}}}}}}_{{mem}}$ is within contact distance of a node on the neighboring cell (${L}_{g,i}\le {L}_{J}$). If true, activate an adhesion element (${\kappa }_{J,{ij}}={\kappa }_{b}{c}_{b,{ij}}{A}_{0}$).
4. At every time point assess if the cadherin bond force within any adhesion element ${{{{{{{{\boldsymbol{E}}}}}}}}}_{{ij}}\in {{{{{{{{\boldsymbol{E}}}}}}}}}_{{adh}}$ has exceeded the critical rupture force (${F}_{b,{ij}}\ge {F}_{{crit}}$). If true, remove the adhesion element (${\kappa }_{J,{ij}}\to 0$).
5. When the simulation time reaches ${t}_{{\max }}$ end the simulation.

By combining and solving the ordinary differential equations (Eqs. 2–4) in conjunction with network force balance, we attain a numerical solution for endothelial monolayer dynamics.

### Statistics and Reproducibility

A two-tailed Student’s t-test was used when comparing difference between two groups. In bar plots, the error bars represent standard deviation and the bar height represents the mean. Observations in PREM images were verified across ten independent experiments for untreated or vehicle-treated samples, two experiments of CK-666-treated cultures and seven experiments of Y-27632 or blebbistatin-treated samples. VE-cadherin was immunolabeled in all PREM experiments. Myosin II immunogold labeling together with VE-cadherin was performed in two independent experiments. Immunofluorescence staining of myosin IIA and F-actin using phalloidin was repeated in two independent experiments. Live cell imaging of cells expressing F-actin reporter (Ftractin or Lifeact) and VE-cadherin was repeated using seven independent experiments for untreated or vehicle-treated cells, two independent experiments for CK-666-treated cells and one experiment for Y-27632 cells. Live cell imaging of cells expressing VE-cadherin and MYL9 was repeated in three independent experiments. For regional analysis of monolayers, data was obtained from three independent experiments.

### Reporting summary

Further information on research design is available in the Nature Portfolio Reporting Summary linked to this article.

## Supplementary information

Supplementary Information

Reporting Summary

Supplementary Movie 1

Supplementary Movie 2

Supplementary Movie 3

Supplementary Movie 4

Supplementary Movie 5

Supplementary Movie 6

Supplementary Movie 7

Supplementary Movie 8

Supplementary Movie 9

Supplementary Movie 10

Supplementary Movie 11

Supplementary Movie 12

Description of Additional Supplementary Files

## Data Availability

Data supporting the findings of this study are available within the article, Supplementary Information and Source Data, and are also available from the corresponding author on request. Source data are provided with this paper.

## Source data

Source Data

## Code availability

MATLAB files used in this work are openly available on Github (https://github.com/EoinMcEvoy).
