# Supplementary material for: Feedback between mechanosensitive signaling and active forces governs endothelial junction integrity
Source: Nat Commun. 2022 Nov 19;13:7089. doi: 10.1038/s41467-022-34701-y (PMC9675837; doi:10.1038/s41467-022-34701-y)
Supplement: Supplementary file 1 — Supplementary Information [file 41467_2022_34701_MOESM1_ESM.pdf]

# Supplemental Information

## Feedback between mechanosensitive signaling and active forces governs endothelial junction integrity

*Eoin McEvoy, Tal Sneh, Emad Moeendarbary, Yousef Javanmardi, Nadia Efimova, Changsong Yang, Gloria E. Marino-Bravante, Xingyu Chen, Jorge Escribano, Fabian Spill, José Manuel Garcia-Aznar, Ashani T. Weeraratna, Tatyana M. Svitkina, Roger D. Kamm, Vivek B. Shenoy*

**SI Section S1: Free-energy-based model of cellular contractility.** Here we derive the equations for myosin-dependent cell contractility introduced in our previous work[1], which form the basis for the feedback model proposed in the accompanying manuscript. We consider a one-dimensional contractile element (e.g., a cell) in its quiescent state, represented by an active actomyosin component and a passive elastic cytoskeleton component (Figure 2b). Application of stress $\sigma$ to this contractile element will lead to changes in its strain $\varepsilon$ and the recruitment of myosin motor proteins. The motors are treated as force dipoles whose magnitude per unit volume is given by $\rho_0$ in the quiescent (stress-free) state and $\rho$ in the stressed state (with units Pa). We aim to characterize the change in strain $d\varepsilon$ and contractility $d\rho$ as a function of an applied stress $\sigma$, to which end we consider that the overall free energy of our system should decrease in accordance with the second law of thermodynamics:

$$dU_{mech}(\varepsilon) + dU_{chem}(\rho) + dU_{motor-work}(\rho, \varepsilon) \leq 0, \qquad (S1.1)$$

where the first two terms denote the changes in the mechanical energy and chemical energies per unit volume of the cell, respectively, and the last term is the mechanical work done by the myosin motors which provides the coupling between strain and contractility. The mechanical energy consists of the elastic energy of the passive cytoskeleton and the mechanical work done by an external stress:

$$U_{mech}(\varepsilon) = \frac{K}{2}\varepsilon^2 - \int_0^{\varepsilon} \sigma d\varepsilon, \qquad (S1.2)$$

where $K$ is the elastic modulus of the passive cytoskeleton. In the quiescent state, the stress generated by the motors attached to the cytoskeleton, $\rho_0$, is determined by a balance of binding and unbinding processes that minimize the net chemical free energy. Perturbations of the contractility from this value should lead to an increase in the chemical free energy. However, stress-activated signally pathways lead to additional recruitment of myosin motors due to an increase in phosphorylated Rho or cytosolic calcium[2], which decreases free energy. Thus, stress alters the equilibrium density of myosin motors to a higher value (with associated higher contractility). The change in chemical free energy can then be written as

$$dU_{chem}(\rho) = \beta(\rho - \rho_0)d\rho - \alpha\sigma d\rho, \qquad (S1.3)$$

where the first term ensures that cell contractility in the absence of cell stress is the quiescent value. The second term represents the chemo-mechanical coupling, wherein stress favors the recruitment of molecular motors, leading to a reduction in free energy. The coupling parameters $\beta$ and $\alpha$ are related to the molecular mechanisms that regulate the engagement of motors and the stress-dependent signaling pathways, respectively. The work done by the motors as the deform the cytoskeleton is given by $U_{motor-work} = \rho\varepsilon$. Through this term, the chemical

energy associated with myosin recruitment and ATP hydrolysis can be converted into mechanical work. Advancing on Eqn S1.1, the total free energy is then

$$U(\rho,\varepsilon)=\frac{K}{2}\varepsilon^2-\int_0^{\varepsilon}\sigma d\varepsilon+\frac{\beta}{2}(\rho-\rho_0)^2-\alpha\int_0^{\rho}\sigma d\rho+\rho\varepsilon\,. \tag{S1.4}$$

To explore the dynamics of cell contractility, it is useful to consider the time variation of the total free energy, which can be written as:

$$\frac{dU(\rho,\varepsilon)}{dt}=\frac{\partial U}{\partial\rho}\frac{\partial\rho}{\partial t}+\frac{\partial U}{\partial\varepsilon}\frac{\partial\varepsilon}{\partial t}\le 0, or \tag{S1.5}$$

$$\frac{dU(\rho,\varepsilon)}{dt}=(\varepsilon+\beta(\rho-\rho_0)-\alpha\sigma)\frac{\partial\rho}{\partial t}+(K\varepsilon+\rho-\sigma)\frac{\partial\varepsilon}{\partial t}\le 0\,. \tag{S1.6}$$

The rate of change of cell contractility can then take the form

$$\frac{\partial\rho}{\partial t}=-k_\rho(\varepsilon+\beta(\rho-\rho_0)-\alpha\sigma), \tag{S1.7}$$

and the rate of change of strain the form

$$\frac{\partial\varepsilon}{\partial t}=-k_\varepsilon(K\varepsilon+\rho-\sigma), \tag{S1.8}$$

to ensure that the rate of change of free energy is always negative ($dU/dt\le 0$), where $k_\rho>0$ and $k_\varepsilon>0$. In the quasi-static analysis carried out in the accompanying manuscript, we assume that $k_\varepsilon\to\infty$ but this value could be varied to describe viscous effects that influence the rate of cytoskeletal contraction. Within Eqn S1.7, $\alpha$ describes molecular mechanisms that regulate stress-dependent signaling pathways. We can reframe this parameter as a function of two separate terms: $\alpha_c'=\alpha_c(c_b/(\gamma_c+c_b))$, which relate to signaling mediated by junctional interactions (SFK, RhoA), and $\alpha_0$, which relates stress to junction-independent pathways such as calcium influx through mechanosensitive channels. Thus, Eqn S1.7 can be extended to the following form:

$$\frac{d\rho}{dt}=-k_\rho\left(\varepsilon+\beta(\rho-\rho_0)-\sigma\left(\alpha_c\left(\frac{c_b}{\gamma_c+c_b}\right)+\alpha_0\right)\right), \tag{S1.9}$$

where $c_b$ is the local concentration of bound cadherin proteins that anchor the cytoskeleton and mediate signaling, and $\gamma_c$ denotes the cadherin concentration for half-strength signaling. To understand the limits of the chemo-mechanical feedback parameters, we can determine the equilibrium condition ($\dot{\rho}=\dot{\varepsilon}=0$), such that $\varepsilon=(\sigma-\rho)/K$ from Eqn S1.8 and $\rho=\rho_0+(\alpha_c'+\alpha_0)/\beta-\varepsilon/\beta$ from Eqn S1.7. Through substitution, we can rephrase the cellular contractility and strain in terms of stress only, whereby:

$$\rho=\frac{K\beta}{K\beta-1}\rho_0+\left(\frac{(\alpha_c'+\alpha_0)K-1}{K\beta-1}\right)\sigma, \qquad and \tag{S1.10}$$

$$\varepsilon = -\frac{\beta}{K\beta - 1}\rho_0 + \frac{\beta - (\alpha_c' + \alpha_0)}{K\beta - 1}\sigma. \qquad (S1.11)$$

We aim to assess whether any parameter choices can lead to instabilities. In this case, clearly both the contractility and strain diverge when $K\beta = 1$. For stability, the chemical "stiffness" parameter, $\beta$, must satisfy the condition $\beta > 1/K$. Further, from a physical perspective, both contractility and strain should increase with increasing force which determines that $(\alpha_c' + \alpha_0) > 1/K$ (via Eqn S1.10) and $\beta > (\alpha_c' + \alpha_0)$ (via Eqn S1.11). However, the value of $\alpha_c'\ (c_b)$ can range between 0 and $\alpha_c$, depending on the concentration of cadherin bonds. Therefore, the limits for the chemo-mechanical parameters are determined to be $\beta > (\alpha_c + \alpha_0)$ and $\alpha_0 > 1/K$ to ensure a stable and physically meaningful model.

**SI Section S2: Cadherin transport (exo-/endocytosis).** The density of unbound cadherin $c_u$ on the membrane is not necessarily fixed, but can change as dependent on active transport processes. Cadherin proteins are transported from within the cell to the membrane through exocytosis, a process we may consider to occur at rate $k_{exo}$. Conversely, endocytosis is the process of moving materials from the membrane into the cell[3], occurring at rate $k_{endo}$. Initially neglecting cell-cell contact phenomena, a simple kinetic equation to describe the flux of cadherin $\dot{n}_c$ between the cytosol and an adhesion site may be given by:

$$\dot{n}_c = -k_{endo} c_u^v + k_{exo} c_c^v \tag{S2.1}$$

where $c_c^v$ is the volumetric concentration of cadherin in the cytosol, $b$ is the membrane thickness, and $c_u^v = c_u/b$ provides the volumetric concentration of unbound cadherin in the membrane. While exocytosis is considered to operate continuously[4], active transport from the membrane is known to be mechanosensitive; low junction tension promotes cadherin loss by endocytosis[5] and inhibition of myosin activity decreases cadherin density at junctions[6]. Thus, we may consider the endocytosis rate coefficient to be stress-dependent such that:

$$k_{endo} = k_e \left(1 - \frac{\sigma_J}{\gamma_e + \sigma_J}\right), \tag{S2.2}$$

where parameter $\gamma_e$ controls the sensitivity of endocytosis processes to junction stress $\sigma_J$. For illustration purposes we can simplify further by assuming a single governing rate for the transport processes $k_{exo} = k_e$, leading to a reduced form:

$$\dot{n}_c = -k_e \left( c_u^v \left(1 - \frac{\sigma_J}{\gamma_e + \sigma_J}\right) - c_c^v \right). \tag{S2.3}$$

With addition of the catch/slip model and main framework, the change in the density of unbound cadherin at a junction can be expressed as:

$$\frac{dc_u}{dt} = -k_e \left( c_u^v \left(1 - \frac{\sigma_J}{\gamma_e + \sigma_J}\right) - c_c^v \right) - \left( k_a c_u - k_0 \left( \exp\left(\frac{F_b}{F_s} - \phi_s\right) + \exp\left(\phi_c - \frac{F_b}{F_c}\right)\right) c_b \right). \tag{S2.4}$$

Here, by changing the sensitivity parameter $\gamma_e$ we can regulate the balance between exo- and endocytosis for a local adhesion site over time as dependent on junction stress $\sigma_J$ (Figure S1). With a low stress sensitivity, we see that the maximum cadherin density remains effectively constant during each "rupture and heal" cycle. Reducing $\gamma_e$ increases the sensitivity of endocytosis to stress, thereby promoting increased cadherin protein reinforcement under tension by reducing endocytosis (while exocytosis operates continuously at a fixed rate).

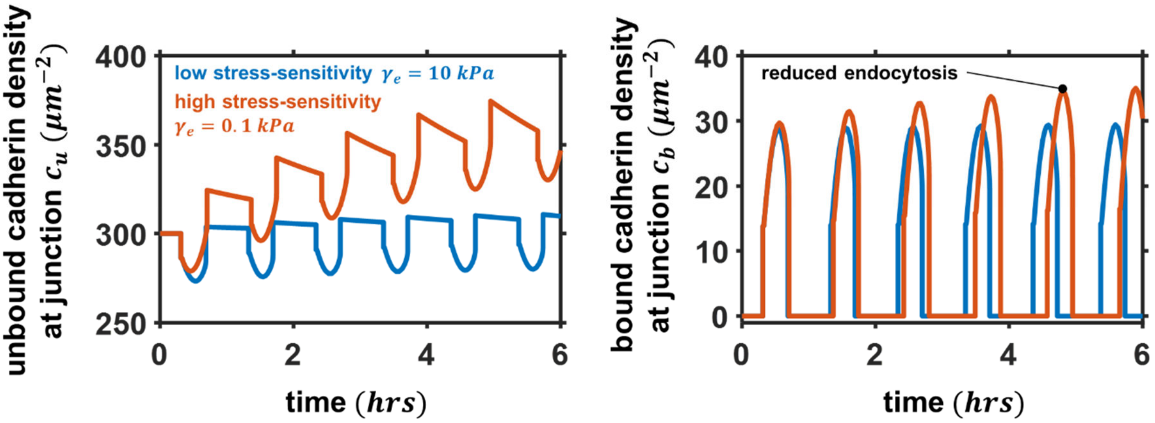


**Figure S1: Predicted unbound and bound density of cadherin within a cell-cell adhesion over a number of cycles with either low sensitivity of endocytosis to stress ($\gamma_e = 10\ kPa$), or a high sensitivity $\gamma_e = 0.1\ kPa$. For these simulations $\alpha_c = 20\ kPa^{-1}$, $\sigma_{P_0} = -3.725\ kPa$, $k_e = 0.0036\ cad.\mu m^{-2}.hr^{-1}.(cad.\mu m^{-3})^{-1}$, and $c_c^v = c_{tot}/b$.**

It is also worth noting that here we implicitly assume a constant cytosolic concentration of cadherin, as we consider the number of cadherin proteins in the cytosol is significantly higher than at the junction (i.e. $N_c \gg N_u$) such that transport has a negligible influence on the cytosolic concentration. However, when applying the framework to a large junction the total number of cadherin proteins in the whole cell $N_{tot}^{cell}$ should be carefully conserved in line with our previous work on focal adhesions[7] by enforcing $N_{tot}^{cell} = c_c^v V_{cell} - \int_{S_{mem}} (c_u + c_b)\, dS$. Here $V_{cell}$ is the volume of the cell body and the second term determines the total number in the membrane (over area $S_{mem}$).

**SI Section S3: Derivation of 1D equilibrium equations.** Initially, considering a cellular element of length $L_{ec}$ and assuming the reference state to be a 1D stress-free connected junction, we can derive an expression for the system stress as governed by mechanical equilibrium. As shown in Figure 2b, we envisage the cell and adhesion as elements in series; this indicates that the stress in the cell and junction is equivalent, i.e. $\sigma_{tot} = \sigma_{ec} = \sigma_J$, where $\sigma_J$ is the stress acting on the junction. Similarly, assuming symmetry in the connected cells, the total change in the length of our system is zero such that $L_{ec}\varepsilon_{ec} + L_J\varepsilon_J = 0$, where $L_J$ is the reference junction length and $\varepsilon_J$ the adhesion strain. This strain will be governed by the effective junction stiffness ($K_j = k_b c_b L_J$), whereby $\varepsilon_J = \sigma_{tot}/(k_b c_b L_J)$. For a closed reference system, we can thus derive an equilibrium expression via:

$$\frac{L_{ec}(\sigma_{tot} - \rho)}{K_{ec}} + L_J\left(\frac{\sigma_{tot}}{k_b c_b L_J}\right) = 0, \quad (S3.1)$$

$$\sigma_{tot}\left(\frac{L_{ec}}{K_{ec}} + \frac{1}{k_b c_b}\right) = \frac{\rho L_{ec}}{K_{ec}}, \quad (S3.2)$$

$$\sigma_{tot} = \frac{\rho L_{ec}}{L_{ec} + \frac{K_{ec}}{k_b c_b}}. \quad (S3.3)$$

As we progress to consider the role of actin polymerization and associated polymerization-induced stress $\sigma_{\mathrm{P}}$ in junction recovery, we evolve this expression (Eqn S3.3) to a reference state with disconnected cells separated by a gap of length $L_g$. The change in cell and junction length therefore leads to a boundary condition $L_{ec}\varepsilon_{ec} + L_J\varepsilon_J = L_g$. Assuming the polymerization-induced stress acts in parallel with the cell passive stress and contractile stress, we expand Eqn S3.1 to find:

$$\frac{L_{ec}(\sigma_{tot} - \rho - \sigma_{\mathrm{P}})}{K_{ec}} + L_J\left(\frac{\sigma_{tot}}{k_b c_b L_J}\right) = L_g, \quad (S3.4)$$

$$\sigma_{tot}\left(\frac{L_{ec}}{K_{ec}} + \frac{1}{\kappa_b c_b}\right) = L_g + \frac{L_{ec}(\rho + \sigma_{\mathrm{P}})}{K_{ec}}, \quad (S3.5)$$

$$\sigma_{tot} = \frac{L_g K_{ec} + L_{ec}(\rho + \sigma_{\mathrm{P}})}{L_{ec} + \frac{K_{ec}}{\kappa_b c_b}}. \quad (S3.6)$$

We note that due to sign convention for compression, the value of $\sigma_{\mathrm{P}}$ will be negative ($\sigma_{\mathrm{P}_0} \leq \sigma_{\mathrm{P}} \leq 0$).

**SI Section S4: Quantitative analysis of cell-cell junctions from PREM.** We analyzed cytoskeleton structures at cell-cell interfaces by platinum replica electron microscopy (PREM), to determine how actin organization differed at cohesive junctions and gaps. Overall, 2000 $\mu m$ of cell-cell contacts under control conditions were analyzed, across 36 images that included 141 gaps and 162 cohesive regions longer than 0.3 $\mu m$ and 31 filopodial bridges with width less than 0.3 μm, which typically form in the course of cell-cell separation[8]. As shown in Table S1, the average length of a cohesive region corresponding to linear adherens junctions was 11.07±15.52 $\mu m$, while the average width of a filopodial bridge corresponding to punctate adherens junctions was 0.20±0.06 $\mu m$. The average length of a gap (as measured along the straight line between the split points) was $2.65 \pm 1.9$ $\mu m$. We identified the average fraction of gaps along cell-cell contacts within a given image to be $19.4 \pm 19.96\%$. Interestingly, branched actin networks were present on average along $95.24 \pm 12.38\%$ of the linear adherens junction length and on average along 51.61±50.8% of punctate adherens junction width. The occurrence of a branched actin network at gaps was quantified as a fraction of gap sides (two sides per gap) associated with any amount of branched actin network, whether it lines up the entire gap side or represented only a small patch. On average, only 18.62±13.26% of gap sides contained branched actin networks.

| | Cohesive regions (>0.3 um in length); n=146 | | Filopodial bridges (<0.3 um in width); n=31 | | Gaps; n=141 (282 sides) | | |
|---|---|---|---|---|---|---|---|
| | Length (um) | Length fraction with branched network (%) | Width (um) | Width fraction with branched network (%) | Length (um) | Length fraction (%) | Fraction of sides with branched networks (%) |
| Average | 11.07 | 95.24 | 0.20 | 51.61 | 2.65 | 19.40 | 18.62 |
| St Dev | 15.52 | 12.38 | 0.06 | 50.80 | 1.90 | 19.96 | 13.26 |
| Max | 79.66 | 100.00 | 0.30 | 100.00 | 11.38 | 68.33 | 100.00 |
| Min | 0.30 | 0.00 | 0.10 | 0.00 | 0.35 | 0.00 | 0.00 |

**Table S1: Quantitative analysis of PREM images.**

**SI Section S5: An analytical analysis of the stress concentration at triple-cell junctions.** We consider a triple-cell junction with a geometry as shown by Figure S2a. We assume both the cell cytoskeleton and adhesion adopt angular shapes such that the polar coordinate $(r,\theta)$ can be conveniently applied. The cell sectors are interconnected with adhesion sectors (represented by boundary lines)[9]. For illustration purposes, we assume that both the cell and adhesions are linear elastic. In either sector, the stress fields $\sigma_r$, $\sigma_\theta$, $\tau_{r\theta}$ and the displacement fields $u_r$, $u_\theta$ in the cells or the adhesions should follow[10]:

$$\sigma_r = -\lambda r^{\lambda-1}[c_1\cos(\lambda+1)\theta + c_2\sin(\lambda+1)\theta \\ +(\lambda-3)(c_3\cos(\lambda-1)\theta + c_4\sin(\lambda-1)\theta)] \qquad (S5.1)$$

$$\sigma_\theta = \lambda r^{\lambda-1}[c_1\cos(\lambda+1)\theta + c_2\sin(\lambda+1)\theta \\ +(\lambda+1)(c_3\cos(\lambda-1)\theta + c_4\sin(\lambda-1)\theta)] \qquad (S5.2)$$

$$\tau_{r\theta} = \lambda r^{\lambda-1}[c_1\sin(\lambda+1)\theta - c_2\cos(\lambda+1)\theta \\ +(\lambda-1)(c_3\sin(\lambda-1)\theta - c_4\cos(\lambda-1)\theta)] \qquad (S5.3)$$

$$u_r = -\frac{r^\lambda}{2\mu}[c_1\cos(\lambda+1)\theta + c_2\sin(\lambda+1)\theta \\ +(\lambda-\kappa)(c_3\cos(\lambda-1)\theta + c_4\sin(\lambda-1)\theta)] \qquad (S5.4)$$

$$u_\theta = -\frac{r^\lambda}{2\mu}[c_1\sin(\lambda+1)\theta - c_2\cos(\lambda+1)\theta \\ +(\lambda+\kappa)(c_3\sin(\lambda-1)\theta + c_4\cos(\lambda-1)\theta)] \qquad (S5.5)$$

Here, $\mu$ is the shear modulus and $\kappa = (3-\nu)/(1+\nu)$, $\nu$ being the Poisson's ratio. $c_i(i = 1,2,3,4)$ are constants and $\lambda$ is a variable[9] that controls how stresses scale with the distance to the junction. Note that the mechanical properties $(\mu,\nu)$ and different sets of coefficients $c_i(i = 1,2,3,4)$ will be different for either the cell or adhesion sector. At the cell-adhesion interface, the stress and displacement must satisfy the continuity condition

$$\sigma_\theta^+ = \sigma_\theta^-, \tau_{r\theta}^+ = \tau_{r\theta}^-, u_r^+ = u_r^-, u_\theta^+ = u_\theta^-. \qquad (S5.6)$$

Here the superscripts + and – denote the two sides of the cell-adhesion boundary. With the representation of displacement and stresses, the singularity at the cell junction can be investigated. We consider a case where three cells form a triple cell junction with 3-fold symmetry ($\alpha = 2°, \phi = 118°$) as shown by Figure S2a. By taking advantage of the symmetry, the problem is reduced to Figure S2b. To satisfy the symmetry, on boundary 1 and 2, $u_\theta = 0, \tau_{r\theta} = 0$. Combining the boundary conditions with Eqns S5.1-S5.5, we obtain a homogeneous linear equation

$$\boldsymbol{Ac} = \boldsymbol{0}, \qquad (S5.7)$$

where the vector $\boldsymbol{c} = [c_1^{cell} \quad c_2^{cell} \quad c_3^{cell} \quad c_4^{cell} \quad c_1^{cad} \quad c_2^{cad} \quad c_3^{cad} \quad c_4^{cad}]^T$. $c_i^{cell}(i = 1,2,3,4)$ and $c_i^{adh}(i = 1,2,3,4)$ denote the four undetermined constants $c_i$ in Eqns S51-S5.5 for the cell and adhesion, respectively. Defining $\gamma = \frac{\phi+\alpha}{2}$, the 8 by 8 matrix $\boldsymbol{A}$ can be written as

$$\begin{bmatrix} 0 & 0 & 0 & 0 & 0 & -1 & 0 & -\lambda-\kappa^{cad} \\ 0 & 0 & 0 & 0 & 0 & -1 & 0 & -\lambda+1 \\ \sin(\lambda+1)\gamma & -\cos(\lambda+1)\gamma & (\lambda+\kappa^{cell})\sin(\lambda-1)\gamma & -(\lambda+\kappa^{cell})\cos(\lambda-1)\gamma & 0 & 0 & 0 & 0 \\ \sin(\lambda+1)\gamma & -\cos(\lambda+1)\gamma & (\lambda-1)\sin(\lambda-1)\gamma & -(\lambda-1)\cos(\lambda-1)\gamma & 0 & 0 & 0 & 0 \\ 1 & 0 & \lambda+1 & 0 & -\cos(\lambda+1)\frac{\phi}{2} & -\sin(\lambda+1)\frac{\phi}{2} & -(\lambda+1)\cos(\lambda-1)\frac{\phi}{2} & -(\lambda+1)\sin(\lambda-1)\frac{\phi}{2} \\ 0 & -1 & 0 & 1-\lambda & -\sin(\lambda+1)\frac{\phi}{2} & \cos(\lambda+1)\frac{\phi}{2} & -(\lambda-1)\sin(\lambda-1)\frac{\phi}{2} & (\lambda-1)\cos(\lambda-1)\frac{\phi}{2} \\ \mu^{cad} & 0 & \mu^{cad}(\lambda-\kappa^{cell}) & 0 & -\mu^{cell}\cos(\lambda+1)\frac{\phi}{2} & -\mu^{cell}\sin(\lambda+1)\frac{\phi}{2} & -\mu^{cell}(\lambda-\kappa^{cad})\cos(\lambda-1)\frac{\phi}{2} & -\mu^{cell}(\lambda-\kappa^{cad})\sin(\lambda-1)\frac{\phi}{2} \\ 0 & -\mu^{cad} & 0 & -\mu^{cad}(\lambda+\kappa^{cell}) & -\mu^{cell}\sin(\lambda+1)\frac{\phi}{2} & \mu^{cell}\cos(\lambda+1)\frac{\phi}{2} & -\mu^{cell}(\lambda+\kappa^{cad})\sin(\lambda-1)\frac{\phi}{2} & -\mu^{cell}(\lambda+\kappa^{cad})\cos(\lambda-1)\frac{\phi}{2} \end{bmatrix} \quad (S5.8)$$

Since Eqn S5.7 is homogeneous, for there to be a non-trivial solution $\boldsymbol{c}$, the determinant of $\boldsymbol{A}$ must vanish ($|\boldsymbol{A}| = 0$). Solving this equation for typical parameters[1] given in Table S2, we find $\lambda - 1 = -0.37$. Note that $\sigma_r \sim r^{\lambda-1}$ and $\sigma_\theta \sim r^{\lambda-1}$ both scale with $r^{\lambda-1}$, therefore a value of $0 < \lambda < 1$ results in infinitely large stresses near the junction as $r \to 0$. This result shows that a symmetric triple cell junction is inherently unstable with a stress singularity at the junction.

| Symbol | Physical meaning | Value |
|---|---|---|
| $\mu^{cell}$ | Cell shear modulus | 1kPa |
| $\mu^{adh}$ | Adhesion effective shear modulus | 0.01kPa |
| $\nu^{cell}$ | Cell Poisson's ratio | 0.3 |
| $\nu^{adh}$ | Adhesion effective Poisson's ratio | 0.3 |

**Table S2. Cell and adhesion mechanical properties.**

We further explore how $\lambda$ can be influenced by the stiffness of cells and adhesions by finding the root of $|\boldsymbol{A}| = 0$ as we vary the ratio $\mu^{adh}/\mu^{cell}$. As shown in Figure S2, we find that the minimum root to $|\boldsymbol{A}| = 0$ always lies in the range $0 < \lambda < 1$ when the adhesions and the cells have stiffness mismatch ($\mu^{adh}/\mu^{cell} < 1$). The stresses become more singular as the $\mu^{adh}/\mu^{cell}$ ratio deviates from 1 (Figure S2c). Note when $\mu^{adh}/\mu^{cell} > 1$, $|\boldsymbol{A}| = 0$ has solutions with $\lambda > 1$ [10], which will not introduce a singularity (Figure S2d). In such case, only with appropriate loading and boundary conditions, can the singularity arise. To further confirm whether the singularity can occur when there is stiffness mismatch ($\mu^{adh}/\mu^{cell} < 1$), we built finite element models (FEM) and assessed the stress near the triple-cell junction. When we apply an isotropic contractile stress that mimics an active endothelial cell contraction, we find

that the singularity arises at the triple-cell junction. The singularity exponent obtained from FEM agrees excellently with the analytical analysis for $\mu^{adh}/\mu^{cell} < 1$ (Figure S2c). These results suggest that the stress singularity was due to the mismatch of the cell and adhesion moduli.

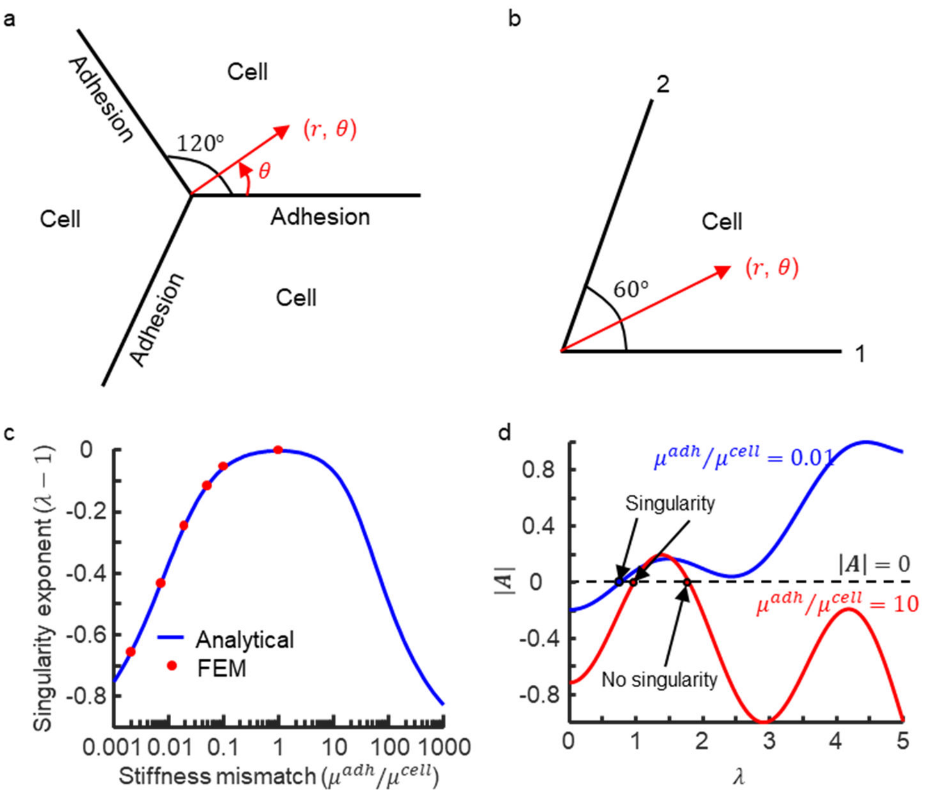


**Figure S2: a) The triple cell junction geometry. We assume that around the junction the cell and cadherin-mediated adhesion follow a 3-fold symmetry. The cell spans an angle of $\phi = 118°$ and the adhesions span an angle of $\alpha = 2°$; b) The repeating unit of the triple cell junction; c) The minimum singularity exponent as a function of the adhesion-cell stiffness mismatch. The singularity vanishes when the adhesion and the cells have the same stiffness ($\mu^{adh}/\mu^{cell} = 1$); d) $|A|$ as a function of $\lambda$ for $\mu^{adh}/\mu^{cell} = 0.01$ *or* 10. A $\lambda > 1$ solution can also be found for $\mu^{adh}/\mu^{cell} = 10$, indicating that the singularity may not occur.**

## SI Section S6: Model parameters

| Parameter | Description | Value |
|---|---|---|
| $\rho_0$ | motor density in quiescent state ($kPa$) | 1 [1,11] |
| $\alpha_0$ | chemo-mechanical feedback parameter (junction independent) ($kPa^{-1}$) | 3 |
| $\alpha_c$ | chemo-mechanical feedback parameter (junction dependent) ($kPa^{-1}$) | 17 |
| $\beta$ | chemical 'stiffness' ($kPa^{-1}$) | 27.7 [1,11] |
| $K_{ec}$ | passive cytoskeletal stiffness ($kPa$) | 1 [1,11] |
| $\gamma_c$ | cadherin concentration for half-strength signaling ($\mu m^{-2}$) | 75 |
| $k_\rho$ | kinetic myosin recruitment constant ($kPa.min^{-1}$) | 0.0024 |
| $c_{tot}$ | total cadherin density at an adhesion site ($\mu m^{-2}$) | 300 [12] |
| $F_s$ | slip force ($pN$) | 16.8 [14] |
| $F_c$ | catch force ($pN$) | 3.47 [14] |
| $\phi_s$ | dimensionless parameter for the slip behavior | $-1.778$ [14] |
| $\phi_c$ | dimensionless parameter for the catch behavior | 2.942 [14] |
| $k_{\mathrm{on}}$ | cadherin binding rate ($s^{-1}$) | 1.2 [14] |
| $\kappa_b$ | effective cadherin stiffness ($nN.\mu m^{-1}$) | 0.5 |
| $F_{crit}$ | bond rupture force ($pN$) | 22 [13] |
| $\sigma_{\mathrm{P}_0}$ | max polymerization-induced stress ($kPa$) | $-4.725$ |
| $k_{\mathrm{P}}$ | kinetic actin polymerization constant ($min^{-1}$) | 0.15 |
| $\gamma_{\rho}$ | myosin density for half-strength antagonism ($kPa$) | 1.75 |
| $K_{mem}$ | membrane element stiffness (Pa) | 100 |
| $R_0$ | reference cell radius ($\mu m$) | 10 |
| $L_g$ | reference gap distance ($\mu m$) | 10 |

**Table S3: Parameters for chemo-mechanical model.**

The parameters associated with cytoskeletal contractility are confined to previously reported ranges[1,11]; namely the quiescent myosin density $\rho_0 = 1\,kPa$, $\beta = 27.7\,kPa$, and $K_{ec} = 1\,kPa$. The density of cadherin proteins at adherens junctions has been measured as on the order of $10^2$-$10^3\,\mu m^{-2}$ [12], motivating us to confine $c_{tot}$ to a magnitude of $300\,\mu m^{-2}$. Catch-slip model parameters are estimated from the experimental data of Manibog *et al.* (2014), as shown in Figure 1c such that $F_s = 16.8\,pN$, $F_c = 3.47\,pN$, $\phi_s = -1.778$, $\phi_c =$

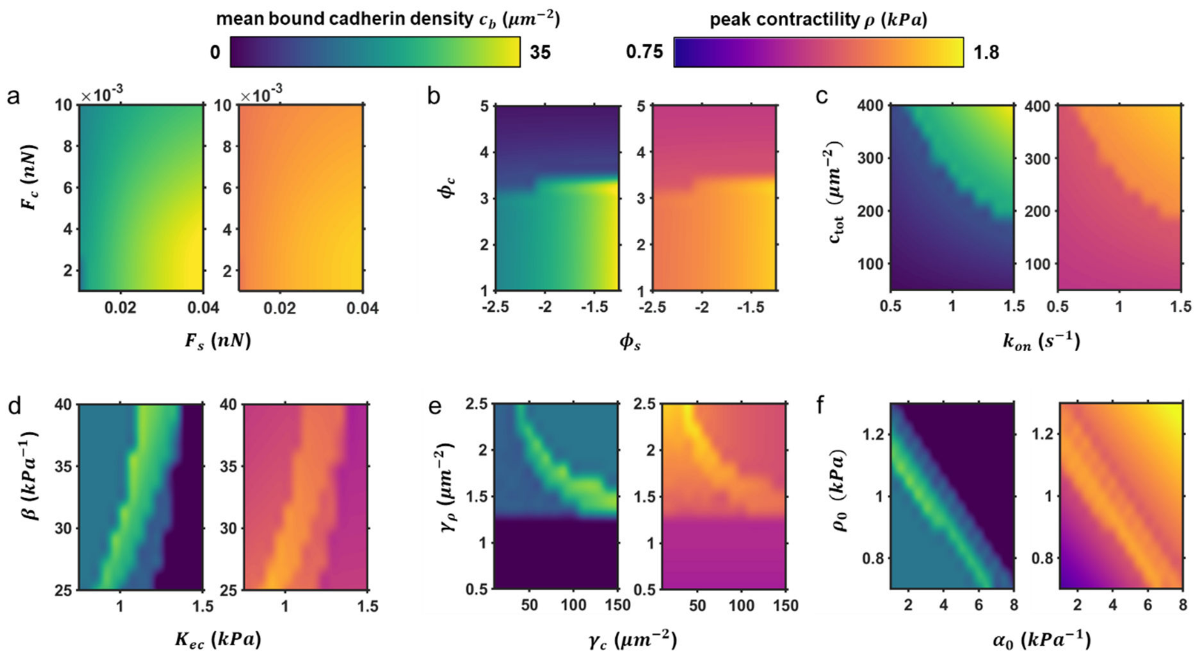


**Figure S3:** ***Sensitivity of model predictions to key parameters*. Changes in predicted bound cadherin density $c_b$ and cell contractility $\rho$ associated with variance in a) catch and slip forces $F_c$ and $F_s$, b) parameters for catch and slip behavior $\phi_c$ and $\phi_s$, c) cadherin binding rate $k_{on}$ and total junction cadherin density $c_{tot}$, d) chemical 'stiffness' $\beta$ and cytoskeletal stiffness $K_{ec}$, e) cadherin dependence parameter $\gamma_c$ and RhoA antagonism parameter $\gamma_\rho$, and f) the junction independent chemomechanical feedback parameter $\alpha_0$ and the quiescent motor density $\rho_0$.**

2.942, and $\kappa_b = 0.5\, nN.\mu m^{-1}$. Manibog *et al.* (2014) also suggest a threshold force of $F_{crit} = 22\, pN$ for the unbinding of a single VE-cadherin bond. The protein binding rate is assumed to be constant and follows Bangasser, Rosenfeld and Odde (2013)[14] whereby $k_{\rm on} = 1.2\, s^{-1}$. We assume a membrane element stiffness of $K_{mem} = 100\, Pa$. The myosin recruitment and polymerization rates ($k_\rho$, $k_{\rm P}$) and the feedback parameters ($\gamma_c$, $\gamma_\rho$) are calibrated to provide good agreement with experimental timescales (Figure 4), with the influence of polymerization parameters shown in Figure S3a-b. The influence of key model parameters on cadherin binding and cell contractility is shown in Figure S3. Briefly, increasing the slip force $F_s$ or reducing the catch force $F_c$ will lead to increased cadherin bond formation and associated contractility and correspondingly, an increase in $\phi_s$ or reduction in $\phi_c$ increases $c_b$ (Figure S3a-b). The density of cadherin bonds reduces when either the total density of cadherin at an adhesion site, $c_{tot}$, or the binding rate, $k_{on}$, is reduced (Figure S3c). A low chemical 'stiffness' parameter, $\beta$, or high cytoskeletal stiffness, $K_{ec}$ can strengthen a junction (Figure S3d) by promoting a high contractility and cell stress, though beyond a critical value a junction may not form unless additional protrusive stress can be generated. A reduction in $\gamma_\rho$ can similarly impede junction formation due to

insufficient polymerization-induced stress (Figure S3d), and a high $\gamma_c$ can push a stable junction into a rupture/heal cycle by promoting excessive contractility. A reduction in either $\alpha_0$ or $\rho_0$ at a stable junction (high cadherin concentration) will reduce contractility $\rho$ and the density of bound cadherin. We also explored the influence of the number of cell protrusions, $m$, on the mean gap size computed during simulations of the cell vertex. As shown in Figure S4, the mean gap area does not change significantly above $m = 12$. Below this value however, the model is highly sensitive to the number of protrusions.

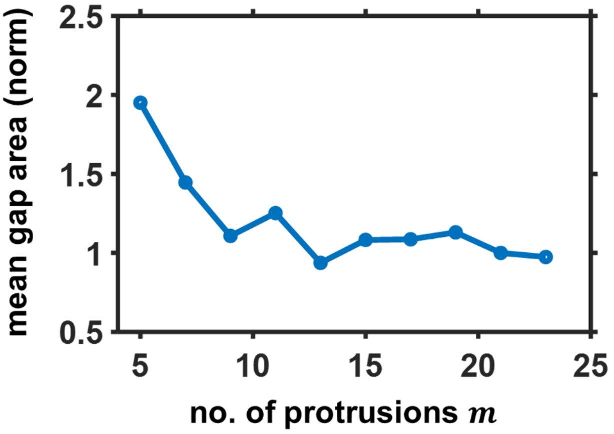


**Figure S4:** ***Influence of protrusion number*** $\mathbf{m}$ ***on vertex simulations*. The mean gap area converges above a critical number of protrusions, $m = 12$. The mean gap area is normalized by the mean area computed at $m = 21$.**

## SI Section S7: Additional Figures

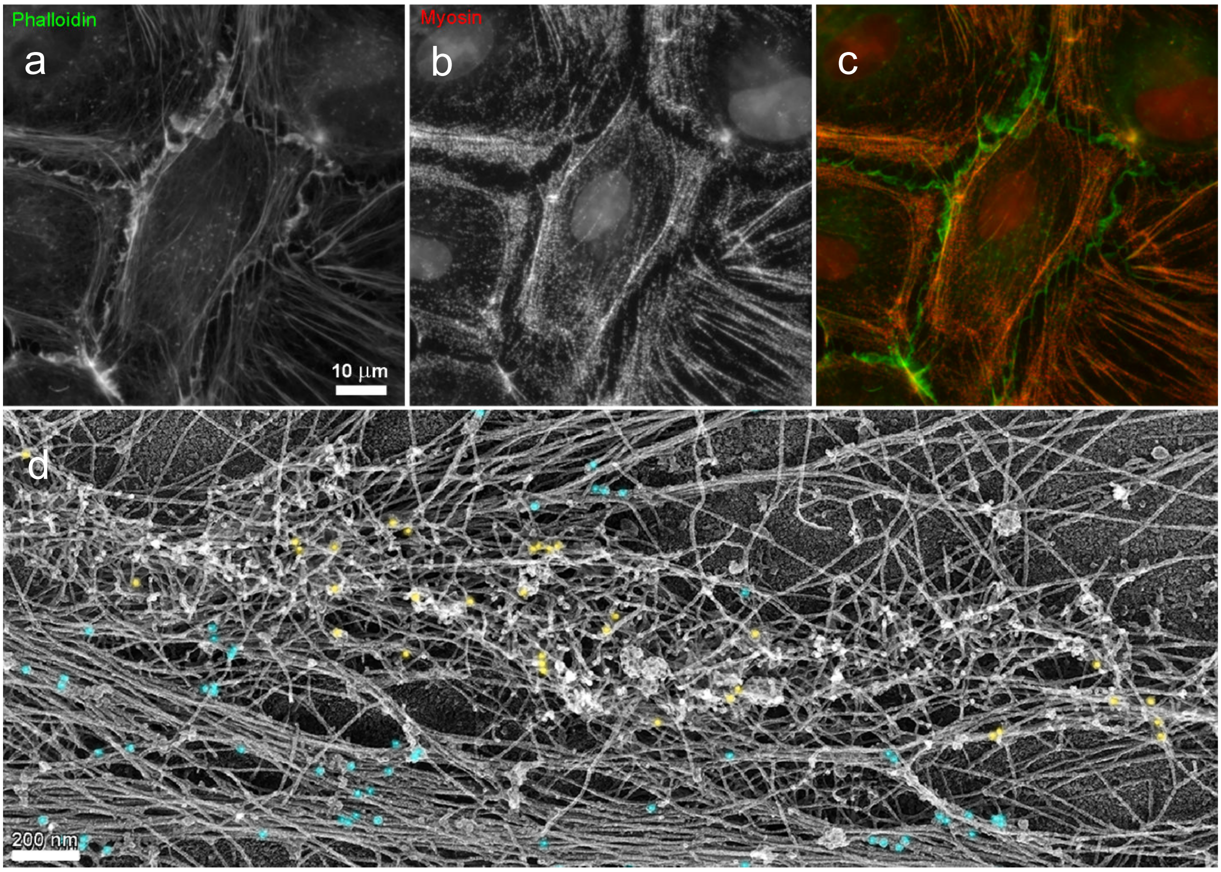


**Figure S5: Staining of a) phalloidin and b) myosin IIA in spread HUVECs shows two actin populations which are clearly distinguishable by morphology in c) merged images. Myosin co-localises with aligned and contracted actin bundles. Scale, 10 $\mu m$; d) PREM image of a cell-cell junction with VE-cadherin immunogold labelled in yellow at the junction with dense branched actin and myosin IIA immunogold labelled in blue on an aligned contractile actin bundle. Scale, 200 nm.**

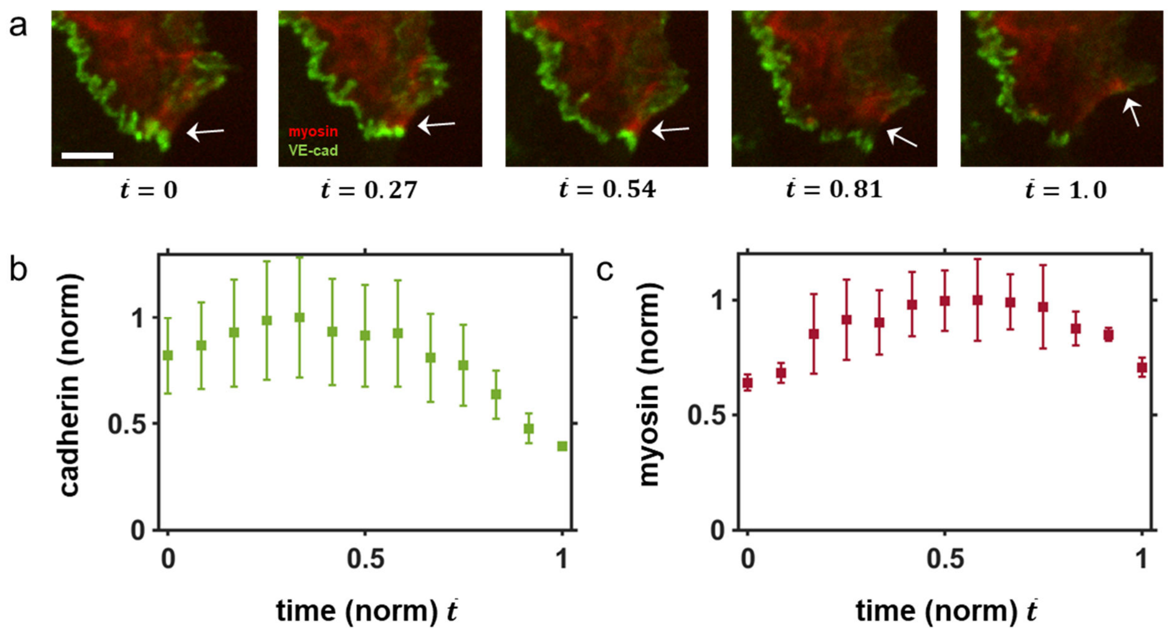


**Figure S6: (a) Feedback between myosin contractility and junction stability in HUVECS over time showing mScarlet-Myl9 (myosin) and GFP-VE-cadherin. The cadherin density initially increases in response to actomyosin contractility but the adhesion subsequently fails as tension continues to increase. Scale, 10 $\mu m$; Local intensity of (b) cadherin and (c) myosin over time normalized to gap opening (t=0) and closing (t=1), (mean $\pm$ s.e.m., $n = 4$ junctions).**

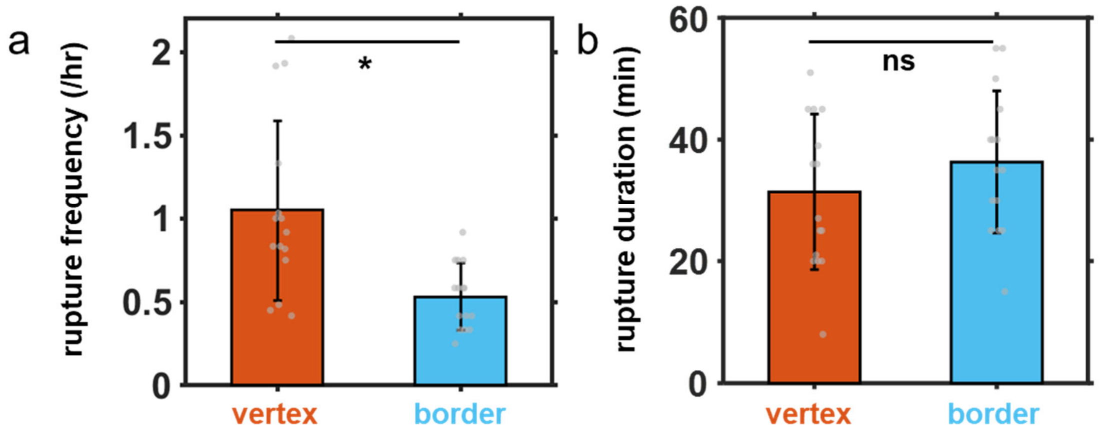


**Figure S7: a) The frequency of rupture and cell-cell vertices is higher than at borders (mean ± s.d., $n = 15$ gaps, P=0.00147); b) The duration of disruption at the vertex and border are similar (mean ± s.d., $n = 15$ gaps). A two-tailed Student's t-test was used when comparing the difference between two groups (*P<0.05).**

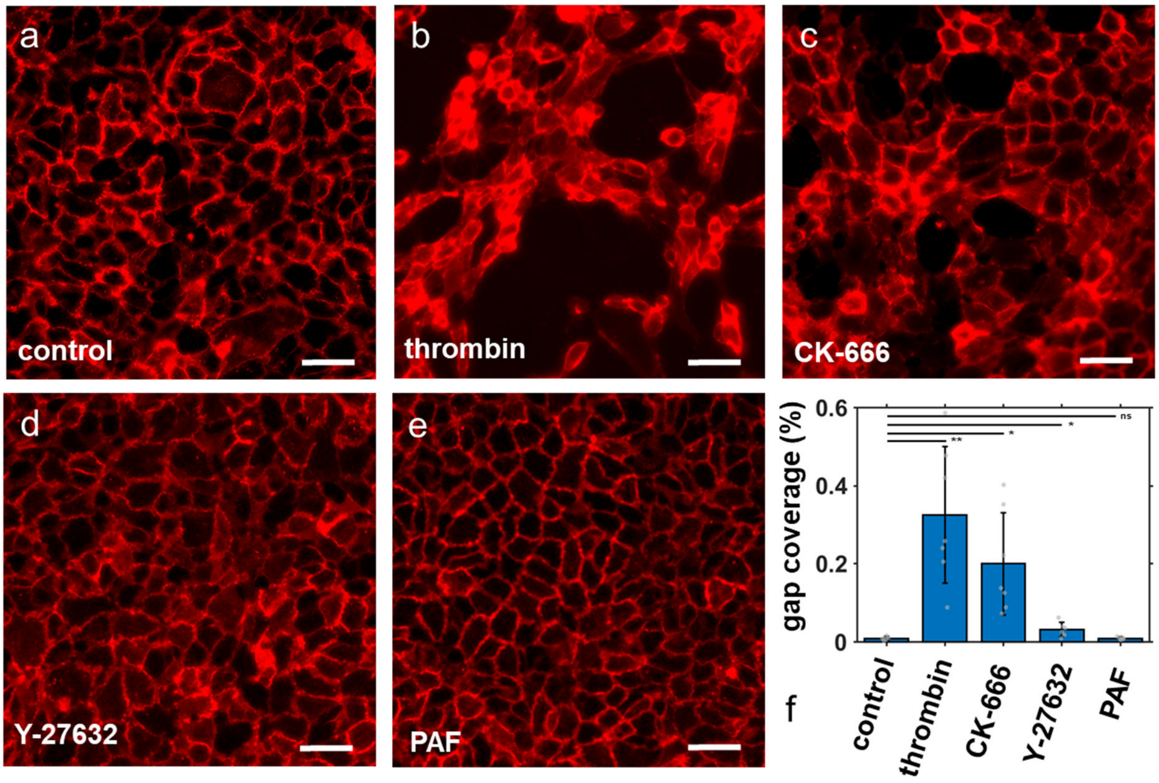


**Figure S8: Organization of HUVEC monolayer under a) control conditions or in response to pharmacological treatment with b) thrombin, c) CK-666, d) Y-27632 or e) PAF. Scale bar, 50 $\mu m$. Red fluorescence shows CD31; f) Percentage gap coverage in treated monolayers (mean $\pm$ s.d., control $n = 5$ regions). A two-tailed Student's t-test was used when comparing the difference between two groups (thrombin $n = 7$ regions, P=0.0026; CK-666 $n = 7$ regions, P=0.0093; Y-27632 $n = 6$ regions, P=0.0269; PAF $n = 6$ regions, P=0.8247; **P<0.005, *P<0.05).**

**SI Section S8: Stochastic modelling of junction dynamics.** The computational model proposed in this paper predicts that periodic gap formation emerges in cycles of cell protrusion, adhesion formation, junction strengthening, and rupture. Repeated gap formation was also observed in our experiments (Supplementary Movies 1 and 11). However, it is worth noting that such in-vitro remodeling is not necessarily periodic, due to inherent randomness in cellular activity. Our model is deterministic in nature, leading to repeated cycles of junction breakdown at calculable timescales. This framework can readily be extended to explore the influence of stochasticity on the regularity of gap formation. We can proceed to modify the chemo-mechanical feedback parameter $\alpha_c$, such that $\alpha_c = \alpha_c'(1 + r)$ within Eqn 3. The parameter $r$ denotes a noise term that follows a normal distribution $r \sim N(0, \Sigma)$, where $\Sigma$ is the variance of the randomness. Simulation of junctional remodeling at a bicellular border indeed reveals that gap formation is less periodic, due to the influence of noisy signaling on actomyosin contractility and cadherin bond stability (Figure S9).

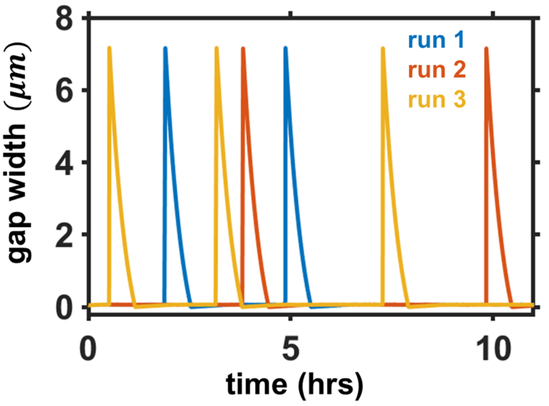


**Figure S9: Simulation of gap dynamic formation at a bicellular junction with stochastic chemo-mechanical signalling feedback ($\alpha_c' = 13\ kPa^{-1}, \Sigma = 0.7$).**

## Supplementary References


1. Shenoy, V. B., Wang, H. & Wang, X. A chemo-mechanical free-energy-based approach to model durotaxis and extracellular stiffness-dependent contraction and polarization of cells. *Interface Focus* **6**, 20150067 (2016).
2. Kimura, K. *et al.* Regulation of myosin phosphatase by Rho and Rho-associated kinase (Rho-kinase). *Science* **273**, 245–248 (1996).
3. De Beco, S., Gueudry, C., Amblard, F. & Coscoy, S. Endocytosis is required for E-cadherin redistribution at mature adherens junctions. *Proc. Natl. Acad. Sci. U. S. A.* **106**, 7010–7015 (2009).
4. Hong, S., Troyanovsky, R. B. & Troyanovsky, S. M. Spontaneous assembly and active disassembly balance adherens junction homeostasis. *Proc. Natl. Acad. Sci. U. S. A.* **107**, 3528–3533 (2010).
5. Troyanovsky, R. B., Sokolov, E. P. & Troyanovsky, S. M. Endocytosis of cadherin from intracellular junctions is the driving force for cadherin adhesive dimer disassembly. *Mol. Biol. Cell* **17**, 3484–3493 (2006).
6. Kale, G. R. *et al.* Distinct contributions of tensile and shear stress on E-cadherin levels during morphogenesis. *Nat. Commun. 2018 91* **9**, 1–16 (2018).
7. McEvoy, E., Deshpande, V. S. & McGarry, P. Free energy analysis of cell spreading. *J. Mech. Behav. Biomed. Mater.* **74**, (2017).
8. Hoelzle, M. K. & Svitkina, T. The cytoskeletal mechanisms of cell-cell junction formation in endothelial cells. *Mol. Biol. Cell* **23**, 310–323 (2012).
9. Sinclair, G. B. Stress singularities in classical elasticity - II: Asymptotic identification. *Appl. Mech. Rev.* **57**, 385–439 (2004).
10. Williams, M. L. Stress singularities resulting from various boundary conditions in angular corners of plates in extension. *J. Appl. Mech.* **19**, 526–528 (1952).
11. Ahmadzadeh, H. *et al.* Modeling the two-way feedback between contractility and matrix realignment reveals a nonlinear mode of cancer cell invasion. *Proc Natl Acad Sci U S A. 2017;114(9)E1617-E26. Epub* **2017/02/16**, E1617--E1626 (2017).
12. Miyaguchi, K. Ultrastructure of the zonula adherens revealed by rapid-freeze deep-etching. *J. Struct. Biol.* **132**, 169–178 (2000).
13. Manibog, K., Li, H., Rakshit, S. & Sivasankar, S. Resolving the molecular mechanism of cadherin catch bond formation. *Nat. Commun.* **5**, 1–11 (2014).
14. Bangasser, B. L., Rosenfeld, S. S. & Odde, D. J. Determinants of maximal force transmission in a motor-clutch model of cell traction in a compliant microenvironment. *Biophys. J.* **105**, 581–92 (2013).
