# Supplementary material for: Feedback between mechanosensitive signaling and active forces governs endothelial junction integrity
Source: Nat Commun. 2022 Nov 19;13:7089. doi: 10.1038/s41467-022-34701-y (PMC9675837; doi:10.1038/s41467-022-34701-y)
Supplement: Supplementary file 2 — Reporting Summary [file 41467_2022_34701_MOESM2_ESM.pdf]

nature research

Corresponding author(s): Vivek B. Shenoy

Last updated by author(s): Oct 24, 2022

Reporting Summary

Nature Research wishes to improve the reproducibility of the work that we publish. This form provides structure for consistency and transparency in reporting. For further information on Nature Research policies, see Authors & Referees and the Editorial Policy Checklist.

Statistics

For all statistical analyses, confirm that the following items are present in the figure legend, table legend, main text, or Methods section.

n/a	Confirmed	
☐	☒	The exact sample size (*n*) for each experimental group/condition, given as a discrete number and unit of measurement
☐	☒	A statement on whether measurements were taken from distinct samples or whether the same sample was measured repeatedly
☐	☒	The statistical test(s) used AND whether they are one- or two-sided<br>*Only common tests should be described solely by name; describe more complex techniques in the Methods section.*
☒	☐	A description of all covariates tested
☒	☐	A description of any assumptions or corrections, such as tests of normality and adjustment for multiple comparisons
☐	☒	A full description of the statistical parameters including central tendency (e.g. means) or other basic estimates (e.g. regression coefficient) AND variation (e.g. standard deviation) or associated estimates of uncertainty (e.g. confidence intervals)
☐	☒	For null hypothesis testing, the test statistic (e.g. *F*, *t*, *r*) with confidence intervals, effect sizes, degrees of freedom and *P* value noted<br>*Give P values as exact values whenever suitable.*
☒	☐	For Bayesian analysis, information on the choice of priors and Markov chain Monte Carlo settings
☒	☐	For hierarchical and complex designs, identification of the appropriate level for tests and full reporting of outcomes
☒	☐	Estimates of effect sizes (e.g. Cohen's *d*, Pearson's *r*), indicating how they were calculated

Our web collection on statistics for biologists contains articles on many of the points above.

Software and code

Policy information about availability of computer code

Data collection	Matlab 2020b was used for simulating cell behavior. Code is freely available in Github repository: https://github.com/EoinMcEvoy
Data analysis	ImageJ was used to measure the frequency, duration, and size of endothelial gaps; NIS Elements for microscopy

For manuscripts utilizing custom algorithms or software that are central to the research but not yet described in published literature, software must be made available to editors/reviewers. We strongly encourage code deposition in a community repository (e.g. GitHub). See the Nature Research guidelines for submitting code & software for further information.

Data

Policy information about availability of data

All manuscripts must include a data availability statement. This statement should provide the following information, where applicable:

- Accession codes, unique identifiers, or web links for publicly available datasets
- A list of figures that have associated raw data
- A description of any restrictions on data availability

Data supporting the findings of this study are available within the article, Supplementary Information and Source Data, and are also available from the corresponding author on request. Source data are provided with this paper.

Field-specific reporting

Please select the one below that is the best fit for your research. If you are not sure, read the appropriate sections before making your selection.

☒ Life sciences ☐ Behavioural & social sciences ☐ Ecological, evolutionary & environmental sciences

For a reference copy of the document with all sections, see nature.com/documents/nr-reporting-summary-flat.pdf

Life sciences study design

All studies must disclose on these points even when the disclosure is negative.

Sample size	No predetermination of sample size was done. Sample size was chosen based on the throughput of the technique used. For each experiment at least three independent replicates were used, and microscope images were acquired at several random locations for each sample. For data analysis, as many gaps as possible were examined to ensure that the 90% confidence interval is sufficiently small (smaller than 5% of average for most cases).
Data exclusions	No data captured was excluded from the subsequent analyses.
Replication	Six endothelial monolayer regions on three separate dishes were experimentally tested, and the results were consistent.
Randomization	No particular randomization strategy was implemented for experiments. For subsampling and choosing the analysed regions complete randomization was performed.
Blinding	For analysis of data regions were chosen randomly without any bias and therefore no blinding procedure was necessary for both experimental and theoretical analysis.

Reporting for specific materials, systems and methods

We require information from authors about some types of materials, experimental systems and methods used in many studies. Here, indicate whether each material, system or method listed is relevant to your study. If you are not sure if a list item applies to your research, read the appropriate section before selecting a response.

Materials & experimental systems

n/a	Involved in the study	
☐	☒	Antibodies
☐	☒	Eukaryotic cell lines
☒	☐	Palaeontology
☒	☐	Animals and other organisms
☒	☐	Human research participants
☒	☐	Clinical data

Methods

n/a	Involved in the study	
☒	☐	ChIP-seq
☒	☐	Flow cytometry
☒	☐	MRI-based neuroimaging

Antibodies

Antibodies used	Alexa Fluor 647 Mouse Anti-Human CD31 (BD Bioscience, 561654) ; Alexa Fluor 647-conjugated PECAM-1 antibody (Hu CD31, BD BioSciences) ; VE-cadherin (BD Biosciences, 610251); Rabbit anti-NMIIA antibody (BT-567); anti-mouse IgG (Jackson ImmunoResearch Laboratories);
Validation	VE-cadherin antibody (Cadherin-5; BD 610251) has been validated by BD Biosciences (more information is available at https://www.bdbiosciences.com/en-ca/products/reagents/microscopy-imaging-reagents/immunofluorescence-reagents/purified-mouse-anti-cadherin-5.610251.). This antibody has been used in several publications (For example, Efimova and Svitkina, 2018, JCB, 217 (5): 1827–1845). Rabbit anti-NMIIA antibody (BT-567) has been previously validated by the Biomedical Technologies (more information is available at https://fnkprddata.blob.core.windows.net/domestic/data/datasheet/BTI/BT-567.pdf). It was also validated by RNAi-mediated knockdown assay (Rai et al., 2017 JBC, 292(8): 3099-3111). All other antibody validations were performed by manufacturers.

Eukaryotic cell lines

Policy information about cell lines

Cell line source(s)	HUVECs were purchased from Lonza (CC-2519)
Authentication	None of the cell lines used were authenticated because they were freshly purchased.
Mycoplasma contamination	Cell lines were not tested for mycoplasma contamination because they were freshly purchased.
Commonly misidentified lines (See ICLAC register)	No commonly misidentified lines were used in this research.
