# Supplementary material for: Feedback between mechanosensitive signaling and active forces governs endothelial junction integrity
Source: Nat Commun. 2022 Nov 19;13:7089. doi: 10.1038/s41467-022-34701-y (PMC9675837; doi:10.1038/s41467-022-34701-y)
Supplement: Supplementary file 15 — Description of Additional Supplementary Files [file 41467_2022_34701_MOESM15_ESM.pdf]

Description of Additional Supplementary Files

Supplementary Movie 1: F-tractin vecadh-contr 01

Supplementary Movie 2: F-tractin vecadh-contr 02

Supplementary Movie 3: Vertex remodeling with GFP tagged VE-CAD

Supplementary Movie 4: Y27632 vecadh LifeAct 01

Supplementary Movie 5: Y27632 vecadh LifeAct 02

Supplementary Movie 6: CK-666 Ftractin vecadh

Supplementary Movie 7: CK-666 LifeAct vecadh

Supplementary Movie 8: Simulated two-cell boundary dynamics with chemo-mechanical feedback α_c = 20 kPa^{-1} and maximum polymerization-induced stress σ_{P0} = −3.725 kPa

Supplementary Movie 9: Simulated endothelial vertex dynamics with chemo-mechanical feedback α_c = 17 kPa^{-1} and maximum polymerization-induced stress σ_{P0} = −4.725 kPa

Supplementary Movie 10: Simulated endothelial vertex dynamics with high chemomechanical feedback α_c = 20.5 kPa^{-1} and maximum polymerization-induced stress σ_{P0} = −4.725 kPa

Supplementary Movie 11: Repeated gap formation at the same vertex, with GFP tagged VE-CAD. Time step between images is 22 mins.

Supplementary Movie 12: Myl9 vecadh
